# Examining the role of moral, emotional, behavioural, and personality factors in predicting online shaming

**DOI:** 10.1371/journal.pone.0279750

**Published:** 2023-03-23

**Authors:** Shannon Raine Muir, Lynne Diane Roberts, Lorraine Sheridan, Amy Ruth Coleman

**Affiliations:** School of Population Health, Curtin University, Bentley, Western Australia, Australia; Bruno Kessler Foundation, ITALY

## Abstract

Online shaming, where people engage in social policing by shaming perceived transgressions via the internet, is a widespread global phenomenon. Despite its negative consequences, scarce research has been conducted and existing knowledge is largely anecdotal. Using a correlational online survey, this mixed-method study firstly assessed whether moral grandstanding, moral disengagement, emotional reactivity, empathy, social vigilantism, online disinhibition, machiavellianism, narcissism, and psychopathy predict participants’ (*N* = 411; aged 15–78) likelihood to engage in online shaming. Two hierarchical multiple regression analyses revealed these predictors significantly accounted for 39% of variance in online shaming intentions, and 20% of variance in perceived deservedness of online shaming (*f*^*2*^ = .25 and .64 respectively, *p* < .001). A content analysis of an open-ended question offered further insights into public opinions about online shaming. These qualitative findings included the perception of online shaming as a form of accountability, the perceived destructive effects of online shaming, the perceived role of anonymity in online shaming, online shaming as a form of entertainment, online shaming involving ‘two sides to every story’, the notion that ‘hurt people hurt people’, online shaming as now a social norm, and the distinction between the online shaming of public and private figures. These findings can be used to inform the general public and advise appropriate responses from service providers and policy makers to mitigate damaging impacts of this phenomenon.

## Introduction

The use of shaming to mitigate undesirable behaviours online has been evident since the internet was first popularised [1]. Whilst there is no widely accepted definition of online shaming, it can broadly be considered a phenomenon where people engage in social policing by shaming perceived transgressions via social media and other internet technologies [2]. Online shaming can take many forms, with common examples including people ridiculing discriminatory posts on social media, or recording and sharing photos or videos of people breaking social norms (e.g., unsafe driving) in public [1]. Much like other forms of shaming, at the heart of this vigilante behaviour lies expressions of social disapproval, with the outcome usually being remorse from the ‘offender’ and disapproval from peers [3].

While online shaming is largely depicted as having palpable and far-reaching negative impacts [4, 5], the perceived benefits to exposing people or organisations online have also been highlighted. For instance, online shaming affords everyday people a platform where they can voice their disapproval of perceived injustices and a multitude of inappropriate behaviours, where these behaviours may have previously remained unpunished or unacknowledged [4]. Online shaming is said to empower individuals to enforce laws and prosocial behaviours, as well as contribute to overall societal reform, and greater institutional transparency [4, 5].

Online shaming typically involves exposing and distributing the content being shamed, followed by various displays of aggression (e.g., insults, threats) and outbursts [5]. Revealing the shamed individuals’ personally identifiable information as a means of condemnation and punishment is also common, with online aggression sometimes evolving into offline harassment (e.g., stalking, vandalising property) afterwards [2, 4]. Whilst one person may have little influence, the collective power from many individuals combined can have a devastating and pervasive impact through the rapid and broad transmission of information online, essentially becoming an online mob trial without due process and no locational or geographical restrictions [2, 4].

Ingraham and Reeves [6, p.456] reflect on the destructive impact modern shaming can have, stating, “the police and the courts are often unable to mete out punishments as severe or intimidating as the ostracism, job loss, death threats, and physical attacks that can accompany what Urry (1999) calls our increasingly mediated culture of shame”. Others discuss the isolation that surrounds the shamed individual and the “impulse to cover up and hide” [4, p.95]. There often is no chance for rehabilitation for those who are shamed, especially given the relative permanency and uncertain timeline of punishments in the online world, however those responsible for the mass shaming are said to often not consider or care about this [7]. More broadly, there is also the possibility that online shaming will contribute to generating an oppressive atmosphere in the online world. This may be amplified due to the absence of proper due processes and lack of guaranteed proportionality, potentially resulting in online shaming becoming increasingly likened to vengeance and bullying [4]. At present, research in this area is still in its infancy, with existing discussion regarding online shaming largely driven by the media and journalists rather than evidenced through empirical inquiry.

### Shaming reimagined

The practice of public shaming has an extensive history, with notable examples of state enforced punishments including public whippings, branding, scarlet letters, and the stockade [4, 8]. Although these practices often served as an effective punishment and deterrent for the wider community, this was all but abolished in the nineteenth century due to being considered too cruel, humiliating, and largely inhumane [8]. Public shaming is said to remove the offender’s dignity, and renders them unable to redeem themselves or ever re-enter ‘normal’ society [8, 9].

As for the online world, the proliferation of internet technologies in the twenty-first century (particularly the rise of social networking websites like Facebook, Instagram, and Twitter) has galvanised individuals’ ability to capture and share their day-to-day lives instantly and with ease [10]. This has led to many members of the public engaging in social or peer surveillance as a form of social control [1], with involvement from authority figures, state officials, and mass media no longer a prerequisite of social norm [11]. Instead, individuals today have an unparalleled agency to observe, judge, and reprimand other internet users for perceived moral infractions [6]. This mass online surveillance has had a pivotal role in the resurrection of shaming as a form of punishment in contemporary society. Further, rather than being simply a re-emergence of public shaming on a contemporary medium, the internet has altered the prerequisites and social conditions needed for public shaming to occur. That is, it is no longer necessary for an actual crime to be committed, but now perceived deviations from social norms are enough to warrant public ridicule for the world to see [11].

### Explaining online shaming

It has been argued that online shaming can be a way for individuals to indicate their non-support for certain socially offensive behaviours, effectively seeking social status by signalling to others their own credibility [12]. Labelled virtue signalling in mass media, and termed moral grandstanding in the literature, this factor has been put forward as a theoretical explanation for online shaming [e.g., 13], but has yet to be examined empirically. Similarly, moral disengagement (convincing oneself that certain ethical standards do not apply to them due to perceived extenuating circumstances) has been demonstrated to be a consistent positive predictor of other acts of online aggression (e.g., cyberbullying) previously [14], but currently has not been tested in relation to online shaming specifically. Again based on comparable online behaviours and media reports of online shaming [15], other potentially useful but currently unexamined predictors of online shaming engagement include higher emotional reactivity [16] and lower empathy [17].

Research by Skoric et al [1] suggests people who shame others online often do not perceive themselves to be shaming individuals, but rather view their activities as a way of contributing to society by reinforcing social norms and deterring others from committing the same ‘offence’. Online shaming is also seen as a way of exerting social control or ‘social policing’, with the aim of deterring deviance and fostering group solidarity [15]. It is the reinforcement of social norms and social control that differentiates online shaming from other activities such as cyber-harassment or online bullying [18]. The intention behind online shaming is said to be to identify and punish social norm violations [1], whereas cyberbullying is instead typically considered to be a repeated personal online attack on an individual (making these online behaviours comparable, but ultimately distinct from each other). Online shaming can also be considered a type of social vigilantism, in that individuals are autonomous, acting spontaneously, and free from organisational or state input to restore justice on their own accord [18]. In this sense, online shaming can be seen as a type of positive punishment, with shaming enforced by everyday people, and transpiring as a reaction to individuals’ dissatisfaction with already established methods of punishment (or lack thereof) for certain acts or behaviours [1].

Another proposed contributing factor of online shaming is online disinhibition [15], which is a phenomenon whereby people feel less restrained and are more willing to express themselves freely on the internet (e.g., harsher criticisms, threats) than in person [19]. A multitude of studies have empirically demonstrated increased aggression and hostile behaviours in computer-mediated interactions compared to face-to-face interactions [e.g., 20], which may also contribute to the ‘mob mentality’ often seen online. Similarly, various personality factors (e.g., psychopathy, sadistic traits) have been shown to predict the likelihood of engaging in aggressive online behaviours [21], and likewise may play a role in online shaming. Collectively, it is evident there are numerous possibly fruitful but currently untested avenues of investigation for researchers wishing to explain and predict online shaming.

### Significance

To date, little evidence exists regarding the motivations for online shaming, which is surprising given the effects can be so devasting and uncontrollable. As previously mentioned, a lot of existing studies pose various concepts as explanations for online shaming without actually testing these, and current discussion on the impacts of online shaming is largely anecdotal or media-driven. The online world is fuelled by social norms, however the means through which these norms are regulated evolve rapidly and unpredictably, rendering online shaming an increasingly pervasive and relevant societal problem. Despite this, current legislative attempts to mitigate the negative effects of online shaming have faced harsh criticism. For example, whilst the European Union’s ‘right to be forgotten’ article, which was put forth in the ‘General Data Protection Regulation’ [22], was met with some support, it largely faced disapproval and outrage [23]. Depending on the jurisdiction and the specific case, there may be several viable legal claims for the person being shamed online (e.g., defamation, criminal hate speech, and use of a telecommunications device to abuse, threaten or harass), however the person conducting the shaming is often not identifiable or there may be too many culpable parties to enact legal consequences against [5]. Whilst there have been cases where legal solutions have been afforded, such as successful defamation lawsuits [5], it is still largely unclear what legal actions are available to those who have been subjected to online shaming. It is essential to systematically analyse and disseminate the motivators and underlying trends of online shaming to establish a more comprehensive understanding of this issue. By doing so, the findings can be used by policy makers to inform appropriate legislation, by service providers to advise appropriate responses, and by psychologists and other health professionals when working with victims of online shaming. Further, findings can also be dispersed to the public to encourage discourse based on empirical support rather than just theoretical and anecdotal speculation.

### The present study

The overarching aim of the present study was to expand upon currently scarce understandings of, and provide empirical evidence for, the motivators behind participation in online shaming. In this paper, we firstly present the utility of several moral, emotional, behavioural, and personality factors in predicting online shaming engagement, followed by an exploratory qualitative account of public opinions regarding online shaming. For the quantitative aspect of this research, the following hypothesis was posited: moral grandstanding, moral disengagement, emotional reactivity, social vigilantism, online disinhibition, machiavellianism, narcissism, and psychopathy will be positive predictors, and empathy be a negative predictor, of likelihood to engage in online shaming.

## Method

### Research design

A cross-sectional, correlational design was employed using an online survey (with a small qualitative component embedded via an open ended-question). The quantitative predictors were moral grandstanding, moral disengagement, emotional reactivity, empathy, social vigilantism, online disinhibition, machiavellianism, narcissism, and psychopathy. The criterion was the likelihood to engage in online shaming. Social desirability was also included as a control variable to statistically account for participants providing responses they may deem to be more desirable. The purpose of the qualitative component was to afford participants the opportunity to share additional thoughts about online shaming not captured within the quantitative strand. The qualitative and quantitative findings have been integrated in the discussion section where appropriate, via a weaving narrative approach.

### Participants

A convenience sample of 411 participants (311 women, 96 men, and 4 identifying as another gender) between 15 and 78 years old (*M* = 24.76, *SD* = 9.59) completed this research, with an average of 14.73 (*SD* = 3.30) years of formal education, and almost half (49%) identifying as Caucasian. The majority of respondents were from Australia (77%), were students (75%), and were employed (60%). The most commonly reported religions were none or Atheist (44%), Catholic (15%), and Christian (15%). On average, participants spent approximately six hours online daily (*SD* = 2.86) and approximately three hours on social media daily (*SD* = 2.14). About 20% (*n* = 84) of participants indicated they had been shamed online before, around 62% (*n* = 256) stated they had not been shamed online before, and just over 17% indicated they were not sure (*n* = 71). For those participants who had been shamed online before and elaborated on their experiences, 25% (*n* = 22) reported being shamed about their appearance (e.g., weight, clothing) and 19% (*n* = 17) reflected they were shamed for having a differing opinion (e.g., views on vaccinations, veganism). Additionally, 7% (*n* = 6) disclosed they were shamed for their political views and 2% (*n* = 2) were shamed for their skin colour. Almost 15% (*n* = 60) of participants indicated they had shamed someone else online before, around 75% (*n* = 308) stated they had not shamed someone else online before, and just over 10% indicated they were uncertain (*n* = 43). Approximately 22% (*n* = 13) of these participants reported shaming another for holding opposing or differing views and 19% (*n* = 12) reflected shaming another over a discriminatory post made by that person (e.g., racist posts). Moreover, 10% (*n* = 6) of these participants reported shaming an individual due to their political views and 5% (*n* = 3) stated they shamed another over their appearance.

Recruitment methods included advertising on a social networking website (where snowballing occurred with other users sharing the post onto their pages; *n* = 152) and a university participant pool (*n* = 259). The opportunity to enter a prize draw was also offered to participants not within the university participant pool, with the only inclusion criteria being that participants were at least 14 years old (to meet the minimum age requirements of most social media platforms). An a-priori power analysis using G*Power 3.1 indicates that for multiple regression, a minimum sample of 114 participants was necessary for a medium effect size, with a power of .80 and significance level of .05 [24]. A medium effect size was chosen based on recent comparable research [e.g., 25]. Based on Kline’s [26] criteria of 20 participants per free parameter, a minimum of 180 participants was necessary to adequately test the factor structure of the Online Shaming Scale. Hence, the obtained sample of 411 participants was deemed adequate to achieve meaningful analyses.

### Measures

#### Criterion variable

Previous measures of online shaming have generally used single or few items to assess online shaming behaviours, which impedes statistical testing and reduces variance [e.g., 27]. The Online Shaming Scale was developed by the authors for the purposes of this study and subsequent research to assess participant likelihood of engaging in online shaming. Based on principal axis factoring with promax rotation of an initial pool of 12 items with a randomised half of the sample (*n* = 206), followed by confirmatory factor analysis in EQS with the remaining half (*n* = 205), the final scale features nine items capturing various hypothetical shaming responses and opinions after a perceived social norm is violated online. All items assess level of agreement via a 7-point Likert-type scale ranging from 1 (*strongly disagree*) to 7 (*strongly agree*). Scores are summed to produce a total online shaming score ranging between 9 and 63, with higher scores indicating a higher likelihood of engaging in online shaming. Total scores were also computed for the two subscales: ‘online shaming intention’ (items 3, 4, 7, 8, 10, 12) and ‘online shaming perceived deservedness’ (items 1, 6, 9), which were used separately in subsequent analyses. See S1 Appendix for further details pertaining to the development of this measure.

#### Predictor and scale control variables

Pre-existing measures for predictor variables (moral grandstanding, moral disengagement, emotional reactivity, empathy, social vigilantism, online disinhibition, machiavellianism, narcissism, and psychopathy) and the control variable social desirability are detailed in Table 1. All items in the Moral Grandstanding Scale [28] were adapted so that they only asked about moral beliefs instead of moral and political beliefs combined (e.g., in the item “I hope that my moral/political beliefs cause other people to want to share those beliefs”, “/political” was removed). For the Perth Emotional Reactivity Scale- Short Form [29], only the items relating to negative emotional reactivity were used as positive emotional reactivity was not expected to be related to online shaming. Question 14 in the Social Vigilantism Scale [30, p.22] was adapted from “I frequently consider writing a letter to the editor” to “I frequently consider writing a product or service review” to reflect contemporary media. Question 1 in the Online Disinhibition Scale [31, p.256] was also adapted from “It is easier to connect with others through ICTs than talking in person” to “It is easier to connect with others online than talking in person” to reflect contemporary language.

**Table 1:** Predictor and control variable measures.

Construct/s	Measure	Factors	Items	Responses	Reversed items	Scoring	α	Example items
Moral grandstanding	Moral Grandstanding Scale [28]	2	10	1–7 (strongly disagree to strongly agree)	-	Mean scores from 1–7	.81-.95	“My moral beliefs should be inspiring to others”
Moral disengagement	Propensity to Morally Disengage Scale [32]	8	16	1–7 (strongly disagree to strongly agree)	-	Mean scores from 1–7	.88	“It’s okay to gloss over certain facts to make your point”
Emotional Reactivity	The Perth Emotional Reactivity Scale- Short Form [29]	3	9	1–5 (very unlike me to very like me)	-	Total scores from 9–45	.76-.91	“I tend to get upset very easily”
Empathy	Basic Empathy Scale [33]	2	20	1–5 (strongly disagree to strongly agree)	1, 6, 7, 8, 13, 18, 19, 20	Total scores from 20–100	.85-.91	“My friends’ emotions don’t affect me much”
Social vigilantism	Social Vigilantism Scale [30]	1	14	1–9 (disagree very strongly to agree very strongly)	-	Mean scores from 1–9	.81-.88	“I feel as if it is my duty to enlighten other people”
Online disinhibition	Online Disinhibition Scale [31]	2	11	0–3 (disagree to agree)	-	Total scores from 0–33	.81-.85	“I feel like a different person online”
Machiavellianism, narcissism, and psychopathy	The Short Dark Triad [34]	3	27	1–5 (disagree strongly to agree strongly)	2, 6, 8 (N) 2, 7 (P)	Mean scores for each factor from 1–5	.71-.77	“It’s not wise to tell your secrets” (M) “People see me as a natural leader” (N) “People often say I’m out of control” (P)
Social desirability	Marlowe-Crowne Social Desirability Scale- Adapted Version [35, 36]	1	13	True or false	1, 2, 4, 5, 7, 9, 11, 12	Total scores from 0–13	.74	“I sometimes try to get even rather than forgive”

*Note*. Measures presented here are self-report scales with higher scores indicating higher levels of each construct. All scoring instructions have been followed according to each measures’ respective author guidelines. α = Cronbach’s Alpha; M = machiavellianism; N = narcissism; P = psychopathy.

#### Demographic variables

Single-item questions concerning demographics (age, gender, country of residence, religion, ethnicity, education attainment level, occupation, daily hours spent online, daily hours spent on social media, previously been shamed online before, and previously shamed someone else online before) were also collected to describe the sample and to be assessed as potential control variables.

#### Qualitative question

Participants also had the opportunity to respond to the optional open-ended qualitative question, “is there anything you would like to say about online shaming?”.

## Procedure

Following ethics approval by Curtin University’s Human Research Ethics Committee (approval number: HRE2019-0697), the survey was piloted (i.e., a few individuals were asked to complete the survey first to ensure comprehension) before data collection took place (between January 23 and September 4, 2020) via a 30-minute online survey. Participants were first directed to a participant information sheet and consent form hosted by Qualtrics.com. After providing informed consent (by ticking a box) and completing validity questions to ensure they understood the contents of the survey, participants were presented with the demographic items, quantitative measures (which were randomised), and open-ended question. Afterwards, participants were debriefed and thanked for their time, and information regarding free online resources in the event of distress were provided. Upon completion, participants were redirected to either a page providing them the opportunity to enter a competition to win a gift voucher by submitting their email address, or a page where they could submit their enrolment details if they were from the university participant pool.

Data were downloaded from Qualtrics.com into IBM SPSS Statistics (v25) to be analysed, with qualitative responses transferred into a Microsoft Excel (v16.4) spreadsheet for analysis. While 500 individuals started the survey, 89 were removed from the quantitative analysis as they either did not complete any items for one or more scales, were found to be duplicates (i.e., identical IP addresses with the same answers) or were patterned responses. As such, data from 411 participants were retained for quantitative analysis and 153 qualitative responses for qualitative analysis. After screening and reverse coding, a missing values analysis indicated that data were missing completely at random, with no variable having missing data exceeding 0.5% (Little’s MCAR test: *χ2*(1130) = 1065.98, *p* = .913). Consequently, the expectation-maximisation method was deemed appropriate for replacing missing values [37]. Mean and total scores were computed and categorical variables were dummy coded prior to analysis.

## Results

### Quantitative results

Table 2 provides descriptive statistics and correlations between predictor, criterion, control, and relevant demographic variables.

**Table 2:** Descriptive statistics and correlations between predictor, criterion, control, and demographic variables (*N* = 411).

Variable	Descriptives			Correlations																				
	*M*	*SD*	α	1	2	3	4	5	6	7	8	9	10	11	12	13	14	15	16	17	18	19	20
1. OSS total	23.12	9.17	.84	-																			
2. OSS intention	13.20	6.87	.86	.93**	-																		
3. OSS PD	9.92	3.81	.65	.74**	.43**	-																	
4. Moral grandstanding [28]	3.53	0.81	.79	.43**	.42**	.28**	-																
5. Moral disengagement [32]	2.47	0.86	.89	.47**	.44**	.34**	.35**	-															
6. Emotional reactivity [29]	27.66	8.39	.91	.14**	.09	.19**	.08	.12*	-														
7. Empathy [33]	77.60	10.10	.89	-.24**	-.26**	-.11*	-.07	-.37**	.15**	-													
8. Social vigilantism [30]	5.17	1.10	.85	.45**	.42**	.33**	.58**	.42**	.15**	-.05	-												
9. Online disinhibition [31]	12.34	5.34	.76	.31**	.32**	.16**	.28**	.40**	.26**	-.11*	.33**	-											
10. Machiavellianism [34]	2.77	0.64	.79	.38**	.33**	.31**	.32**	.60**	.18**	-.25**	.45**	.41**	-										
11. Narcissism [34]	2.60	0.58	.72	.25**	.24**	.17**	.29**	.26**	-.06	-.18**	.41**	.15**	.36**	-									
12. Psychopathy [34]	2.04	0.60	.76	.43**	.45**	.24**	.21**	.57**	.19**	-.33**	.30**	.27**	.50**	.37**	-								
13. Social desirability [35, 36]	5.67	2.57	.64	-.13*	-.09	-.15**	-.10*	-.26**	-.35**	-.07	-.23**	-.22**	-.29**	-.10	-.26**	-							
14. Age	24.76	9.59	-	.04	.08	-.07	-.13**	-.22**	-.19**	<-.01	-.07	-.16**	-.09	-.05	-.12*	.17**	-						
15. Gender	-	-	-	-.13**	-.16**	-.04	-.02	-.23**	.12*	.28**	-.09	-.15**	_.15**	-.15**	-.27**	.04	.02	-					
16. Years of education	14.73	3.30	-	.01	.01	-.01	-.05	.11*	-.05	.05	-.03	-.15**	<.01	-.02	-.09	.05	.34**	.03	-				
17. Daily online hours	6.11	2.86	-	.12*	.11*	.09	.03	.12*	.16**	-.07	.07	.11*	.12*	.07	.15**	-.12*	-.18**	-.06	-.02	-			
18. Daily SM hours	3.16	2.14	-	.12*	.12*	.07	.17**	.15**	.21**	.10*	.19**	.21**	.11*	.10*	.10*	-.13**	-.26**	.13**	-.09	.55**	-		
19. Been OS	-	-	-	.03	.06	-.02	.05	-.08	.05	.15**	.06	<.01	-.11*	.11*	.04	-.12*	.09	.08	-.03	.02	.04	-	
20. OS someone	-	-	-	.28**	.29*	.15**	.20**	.12*	.06	.03	.17**	.06	.11*	.11*	.21**	-.17**	.11*	-.07	-.02	.08	.01	.27**	-

*Note*. Gender was coded as 0 = not women, 1 = women. Been online shamed and online shamed someone were coded as 0 = no or unsure, 1 = yes. *M* = mean; *SD* = standard deviation; α = Cronbach’s Alpha; OSS = Online Shaming Scale; PD = perceived deservedness; SM = social media; OS = online shamed.

*p < .05.

**p < .01.

#### Hypothesis testing

To test the overarching quantitative hypothesis, that moral grandstanding, moral disengagement, emotional reactivity, social vigilantism, online disinhibition, machiavellianism, narcissism, and psychopathy will be positive predictors, and empathy will be a negative predictor of likelihood to engage in online shaming, two hierarchical multiple regression analyses (HMRAs) were completed (separate regressions for the online shaming subscales were conducted as differences between subscales in the correlation matrix were noted). The correlation matrix also revealed associations between online shaming and gender, daily online hours, daily social media hours, having shamed someone online before, and social desirability, and therefore these variables were used as additional control variables. All demographic control variables were entered into step one of both regressions, with social desirability entered as another control variable in step two, and the study predictors entered altogether in step three (in no particular order as there was no theoretically driven reason to do so). Assumptions underlying multiple regression were evaluated, with all relevant variables approximating normality except for the online shaming intention subscale, which approximated an L-shaped distribution and had heteroscedastic residuals [37]. Data transformation following guidelines by Tabachnick and Fidell [37] were conducted to remedy this, however this did not alter the overall findings or improve the skewness of the variable, so the original data were retained for analysis. See S2 Appendix for all normality plots. Univariate outliers were reduced to the most extreme non-outlier values plus one unit to maintain rank order [37], and no highly influential multivariate outliers were present. All other assumptions were met, and statistical significance for this hypothesis testing was evaluated at α = .05.

The first HMRA was conducted to assess the proportion of variance in online shaming intentions that could be accounted for by moral grandstanding, moral disengagement, emotional reactivity, empathy, social vigilantism, online disinhibition, machiavellianism, narcissism, and psychopathy (after controlling for gender, daily online hours, daily social media hours, having shamed someone online before, and social desirability). On step 1 of this HMRA, gender, daily online hours, daily social media hours, and having shamed someone online before collectively accounted for a significant 12% of the variance in online shaming intentions, *R*^*2*^ = .12, adjusted *R*^*2*^ = .11, *F* (4, 406) = 14.03, *p* < .001. On step 2, social desirability was added to the regression model, accounting for a non-significant additional < .01% of the variance in online shaming intentions, Δ*R*^*2*^ *=* .00, Δ*F* (1, 405) = .10, *p* = .748. These five variables in combination still explained 12% of the variance in online shaming intentions, *R*^*2*^ = .12, adjusted *R*^*2*^ = .11, *F* (1, 405) = 11.22, *p* < .001. On step 3, moral grandstanding, moral disengagement, emotional reactivity, empathy, social vigilantism, online disinhibition, machiavellianism, narcissism, and psychopathy were added to the regression model, accounting for a significant additional 27% of the variance in online shaming intentions, Δ*R*^*2*^ *=* .27, Δ*F* (9, 396) = 19.60, *p* < .001. All variables in combination explained 39% of the variance in online shaming intentions, *R*^*2*^ = .39, adjusted *R*^*2*^ = .37, *F* (9, 396) = 18.27, *p* < .001. This is a large effect by Cohen’s [38] conventions (*f*^*2*^ = .64).

The second HMRA was conducted to assess the proportion of variance in perceived deservedness of online shaming that could be accounted for by moral grandstanding, moral disengagement, emotional reactivity, empathy, social vigilantism, online disinhibition, machiavellianism, narcissism, and psychopathy (after controlling for gender, daily online hours, daily social media hours, and having shamed someone online before, and social desirability). On step 1 of this HMRA, gender, daily online hours, daily social media hours, and having shamed someone online before collectively accounted for a significant 3% of the variance in perceived deservedness of online shaming, *R*^*2*^ = .03, adjusted *R*^*2*^ = .02, *F* (4, 406) = 3.27, *p* = .012. On step 2, social desirability was added to the regression model, accounting for a significant additional 1% of variance in perceived deservedness of online shaming, Δ*R*^*2*^ *=* .01, Δ*F* (1, 405) = 5.56, *p* = .019. These five variables in combination explained 4% of the variance in perceived deservedness of online shaming, *R*^*2*^ = .04, adjusted *R*^*2*^ = .03, *F* (1, 405) = 3.76, *p* = .002. On step 3, moral grandstanding, moral disengagement, emotional reactivity, empathy, social vigilantism, online disinhibition, machiavellianism, narcissism, and psychopathy were added to the regression model, accounting for a significant additional 16% of the variance in perceived deservedness of online shaming, Δ*R*^*2*^ *=* .16, Δ*F* (9, 396) = 8.70, *p* < .001. All variables in combination explained 20% of the variance in perceived deservedness of online shaming, *R*^*2*^ = .20, adjusted *R*^*2*^ = .17, *F* (9, 396) = 7.17, *p* < .001. This is a medium effect by Cohen’s [38] conventions (*f*^*2*^ = .25). Regression coefficients, squared semi-partial correlations, and confidence intervals for both models are displayed in Table 3. Additionally, see S3 Appendix for a comparison between the current linear regressions and post hoc Ridge regressions as an additional robustness check of the current findings.

**Table 3:** Summary of regression coefficients and squared semi-partial correlations (sr^2^) for two hierarchical regression models predicting online shaming intentions and perceived deservedness (*N* = 411).

	Predictors	OSS intentions					OSS perceived deservedness				
		Coefficient		95% CI Around *B*		*sr* ^2^	Coefficient		95% CI Around *B*		*sr* ^2^
		*B*	β	LB	UB		*B*	β	LB	UB	
Step I											
	Gender	-2.47	-.16	-3.96**	-.98**	.02	-.27	-.03	-1.15	.60	<.01
	Daily online hours	.03	.01	-.23	-.98	<.01	.07	.05	-.09	.22	<.01
	Daily SM hours	.38	.12	.03*	.74*	<.01	.09	.05	-.12	.30	<.01
	OS someone	5.41	.28	3.63**	7.19**	.08	1.54	.14	.50**	2.59**	.02
Step 2											
	Gender	-2.46	-.15	-3.95**	-.97**	.02	-.23	-.03	-1.10	.63	<.01
	Daily online hours	.03	.01	-.23	.30	<.01	.06	.05	.09	.22	<.01
	Daily SM hours	.38	.12	.02*	.73*	<.01	.07	.04	-.14	.28	<.01
	OS someone	5.36	.28	3.56**	7.17**	.07	1.33	.12	.28*	2.39*	.02
	Social desirability	-.04	-.02	-.29	.21	<.01	-.17	-.12	-.32*	-.03*	.01
Step 3											
	Gender	<.01	<.01	-1.35	1.36	<.01	.30	.03	-.57	1.12	<.01
	Daily online hours	.07	.03	-.16	.30	<.01	.08	.06	-.07	.22	<.01
	Daily SM hours	<.01	<.01	-.32	.32	<.01	-.10	-.05	-.30	.11	<.01
	OS someone	3.51	.18	1.93**	5.09**	.03	.72	.07	-.29	1.73	<.01
	Social desirability	.26	.10	.03*	.49*	<.01	.02	.01	-.13	.17	<.01
	Moral grandstanding	1.50	.18	.68**	2.32**	.02	.52	.11	<-.01	1.04	<.01
	Moral disengagement	.92	.11	.01*	1.83*	<.01	.88	.20	.30**	1.47**	.02
	Emotional reactivity	<-.01	<-.01	-.08	.07	<.01	.06	.14	.02*	.11*	.01
	Empathy	-.08	-.12	-.14*	-.02*	<.01	-.01	-.03	-.05	.03	<.01
	Social vigilantism	1.13	.18	.46**	1.80**	.02	.52	.15	.09*	.95*	.01
	Online disinhibition	.16	.12	.04**	.28**	.01	-.05	-.06	-.12	.03	<.01
	Machiavellianism	-.55	-.05	-1.69	.60	<.01	.59	.10	-.14	1.32	<.01
	Narcissism	-.48	-.04	-1.57	.61	<.01	.02	<.01	-.68	.72	<.01
	Psychopathy	2.77	.24	1.57**	3.97**	.03	-.06	-.01	-.83	.71	<.01

*Note*. CI = confidence interval; OSS = Online Shaming Scale; *B* = unstandardised coefficient; β = standardised coefficient; LB = lower bound; UB = upper bound.

*p < .05.

**p < .01

### Qualitative findings

A mixed (conventional and summative) content analysis [39] was used to analyse the open-ended survey question “is there anything you would like to say about online shaming?”. The conventional approach was used to describe emergent categories, with the summative approach used to quantify the frequency of responses within each category [39]. All participant responses were first read in their entirety by one researcher, before open coding was conducted with relevant meaningful responses noted and afterwards assigned a phrase or key word summarising each response. After two rounds of open coding, categories were created and then split, combined, or expanded upon where necessary. The summative aspect involved tallying the frequency with which the final categories occurred in the dataset, with words or phrases with interchangeable meanings also included within this count. To assess inter-rater reliability, approximately 10% of statements were classified as either in support of each category or against by two researchers using IBM SPSS Statistics (v25). Both coders have been trained in and have experience conducting qualitative analysis. An excellent level of inter-rater agreement (κ = .75) was found [40].

Holding people accountable, depicting online shaming as having destructive effects, and perceived anonymity afforded by the online world were the most frequent categories to emerge. Multiple participants also explored online shaming as a form of entertainment, as a phenomenon with multiple perspectives, and as often perpetrated by individuals coming from a place of shame or hurt. Some participants also regarded online shaming as now a social norm, as well as exploring perceived differences between public and private figures being shamed online. The complete findings of this content analysis are displayed in Table 4.

**Table 4:** Content analysis of what participants have to say about online shaming (*n* = 153).

Category	*n*	Description	Example quotes
Accountability	10	Online shaming is deemed acceptable and encouraged in certain settings when undertaken to hold an individual or group of individuals accountable and/or to raise awareness. Some participants appeared to view engaging in online shaming as serving the greater good, as it allows individuals to be informed and hold the perpetuator responsible for their actions online.	“I only participate in shaming behaviour if I know for a fact that they’re acting abusive and needs to be held accountable for it.”
Destructive effects	7	Participants referenced the harmful impacts of online shaming in a general sense but tended to avoid specific details. This may indicate that while there is a level of public awareness, people may not be aware of the specific, tangible consequences of online shaming.	“Its very dangerous and can cause alot of harm”
Anonymity	7	Anonymity provided through being online and the use of private accounts may allow people to feel more comfortable and safe to engage in online shaming. This sense of anonymity may afford people perceived power, which may embolden them to engage in online shaming. Participants directly contrasted online shaming to face-to-face bullying and suggested this would not occur in the latter setting.	“people feel they are anonymous online and feel safe to write evil hurtful things”
Entertainment	6	This category describes online shaming, including the use of memes, as a form of entertainment. Participants also articulated that online shaming and sharing memes may serve as a way to engage with friends and other community members.	“I only online shame them so that other people (who follow my private account) are aware of the things they say and we can laugh about it.”
Two sides to every story	6	While there are two sides to every story, participants articulated that individuals should have the ability to express their opinion without being shamed. Online shaming and freedom of speech appears almost paradoxical; people expressing their opinion without worrying about being shamed is freedom of speech, but opposingly freedom of speech is also having the right to shame someone if you do not agree with them.	“There is always 2 sides to every story and everyone has the right to their own opinion.”
“Hurt people hurt people”	4	Some participants referenced that being shamed online may cause a victim of online shaming to turn into a perpetrator. There is a belief that the perpetrator of online shaming may engage in this behaviour due to insecurities or past experiences with being shamed.	“It is a vicious cycle of people being online shamed and then going on to online shame others”
Social norm	3	Multiple participants referenced that online shaming has become a social norm.	“Online shaming is considered a social norm”
Public versus private figures	3	Participants reflected that individuals are held to different standards, depending on whether they are “public figures” or ‘everyday’ people. Public figures appear to be more readily online shamed, with some suggesting they are more deserving of this, due to having chosen to become a public figure or having a public platform.	“I am quick to call public figures out, as they have chosen a position of influence. With random people, I’m more restrictive.”

*Note*. Single participant responses were able to be coded into multiple categories.

## Discussion

The main purpose of this study was to assess the utility of several currently untested predictors of online shaming, and given this research area is still in its infancy, we also sought to qualitatively capture general public opinions about online shaming. Quantitative results indicated that in combination, moral grandstanding, moral disengagement, emotional reactivity, empathy, social vigilantism, online disinhibition, machiavellianism, narcissism, and psychopathy accounted for approximately two fifths of the variance in intentions to engage in online shaming, and one fifth of the variance in perceived deservedness of online shaming (after controlling for gender, daily online hours, daily social media hours, having shamed someone online before, and social desirability). Some qualitative responses converged with quantitative findings regarding moral disengagement, empathy, social vigilantism, and online disinhibition, whilst other qualitative responses suggested alternative or divergent understandings of online shaming. These findings are contextualised and explored in detail below.

### Contextualising the current findings

Scores for the intentions subscale of the Online Shaming Scale indicated that on average, participants *disagreed* with the notion of intending to shame someone online. Scores for the perceived deservedness subscale were slightly higher, with participants on average only *somewhat disagreeing* that people who were shamed online deserved it. With the two subscales combined, participant total scores for online shaming fell between *disagree* and *slightly disagree*, indicating an overall trend towards not engaging in online shaming. Interestingly, the two subscales of the Online Shaming Scale only had a moderate positive correlation with each other, which combined with the slightly higher perceived deservedness scores, indicates that perhaps while some individuals may believe people to be deserving of being shamed online, they may be less willing to conduct the actual shaming themselves. From a measurement standpoint, this also supports the inclusion of both online shaming subscales rather than just one or the other.

Demographic data indicated that approximately one fifth of participants reported having been subjected to online shaming before, and slightly less than one fifth of participants reported having shamed someone else online before. In comparable research (e.g., exploring online harassment, cyberbullying), whilst some results indicate lower rates, many report higher [see 41–43]. The comparatively lower prevalence of online shaming in the current study may not be due to an actual lack of online shaming occurring, but as echoed previously within the literature [e.g., 1], participants may instead just not label their actions as online shaming. Instead, people may consider their actions to be a social deterrent and a mechanism to bolster accountability, a notion which was also salient within the current qualitative findings. Moreover, previous engagement with online shaming victimisation and perpetration were both only measured using a single item rather than measuring various types online shaming separately, indicating a lack of specificity which may also explain possibly understated rates of online shaming. The notion of online shaming now being considered a common and expected behaviour in society, which is reflected in both previous literature [e.g., 11] and the current qualitative findings, also serves to contrast these lower reports of online shaming. Another noteworthy finding is that prior online shaming had no significant relationship with any other demographic variable, which is interesting considering factors such as the gendered nature of online shaming (e.g., slut shaming, body shaming) often reported in previous literature [e.g., 15, 44]. There was also no significant relationship between online shaming victimisation and perpetration, which also contrasts related claims (i.e., the cybervictimisation to cyberbullying cycle often reported in cyberbullying literature) in similar studies [43], as well as contrasting participant perceptions offered in the current qualitative findings. These inconsistencies between the demographics in the current study and that of previous literature may simply be an indication of sample specific findings, or perhaps due to measurement differences between online shaming studies, or changing trends in online shaming. Regardless, this warrants further investigation into the relationship between demographic differences and online shaming in future research.

Whilst there were no significant associations between intentions to online shame and age, years of education, having been subjected to online shaming before, and social desirability, men and those who spent more hours online and on social media each week were slightly more likely to intend to engage in online shaming. There was also a moderate positive relationship between having shamed someone online before previously and future intentions to online shame. Although this was used as a control variable for subsequent hypothesis testing, previous online shaming actually accounted for the same unique variance in online shaming intentions as the strongest predictor, which echoes the age-old sentiment of the best predictor of future behaviour being past behaviour. As for the second online shaming subscale, there were also no significant associations between perceived deservedness of online shaming and age, gender, years of education, daily hours online and on social media, and having been subjected to online shaming before. However, there was a small positive relationship between perceived deservedness of online shaming and having shamed someone online before previously, as well as a small negative correlation between perceived deservedness and social desirability. As for the predictors in this study, there were significant relationships (ranging from small to large) between all predictors and both online shaming subscales, with the exception of online shaming intentions and emotional reactivity. The predictive utility of these variables are further deconstructed below.

### Moral predictors: Moral grandstanding and moral disengagement

Moral grandstanding and moral disengagement both had moderate positive correlations with both online shaming factors, indicating that participants with a stronger desire to appear morally credible to others and a propensity to convince themselves that ethical standards do not apply to them due to apparent extenuating factors were more likely to engage in online shaming and believe those who are shamed are deserving of this treatment. These findings are consistent with current theoretical musings relating to online shaming [e.g., 12, 13], the cyberbullying literature [e.g., 14], as well as some sentiments expressed by participants in the current qualitative findings. In the regression models, both moral predictors accounted for unique variance in intentions to online shame, however only moral disengagement (and not moral grandstanding) accounted for unique variance in perceived deservedness of online shaming (with moral disengagement being the strongest predictor of perceived deservedness).

### Emotion predictors: Emotional reactivity and empathy

Emotional reactivity had a small positive correlation with perceived deservedness of online shaming, however it did not correlate significantly with intentions to online shame, suggesting that while participants who are more emotionally reactive may be more likely to believe people to be deserving of online shaming, this does not appear to impact their decision to actually engage in online shaming or not. This contradicts the notion of online shaming being a ‘knee-jerk’ reaction, as previously depicted in news media [15], which represents a potential discrepancy between the explanations hyper-publicised news stories provide for online shaming and what is actually occurring. Empathy however, was negatively correlated with both perceived deservedness and online shaming intentions (with the associations being small and small-to-medium in size, respectively), indicating empathy may be a protective factor against both believing people to be deserving of online shaming and intending to online shame. This coincides with studies on related online behaviours [e.g., 17], and is reinforced by participant concerns over the destructive effects of online shaming illustrated within the qualitative findings. In the regressions, emotional reactivity accounted for unique variance in perceived deservedness of online shaming, but not intentions to online shame. For empathy the opposite occurred, accounting for unique variance in intentions to online shame but not perceived deservedness.

### Behavioural predictors: Social vigilantism and online disinhibition

Social vigilantism had medium positive correlations with both deservedness of online shaming and intentions to online shame, indicating that participants with a higher superiority of beliefs and perceived responsibility to propagate these beliefs were more likely to intend to engage in online shaming and believe victims of online shaming to be deserving. This aligns with literature that links online shaming to social vigilantism [e.g., 1, 18], with some current qualitative responses relating to accountability also converging with this notion. Online disinhibition had a moderate positive association with intentions to online shame, and a small positive correlation with perceived deservedness, demonstrating that those who feel more willing to express themselves freely on the internet and experience a lack of restraint online compared to in-person interactions are more likely to intend to online shame and perceive those who are shamed online to be deserving. Online disinhibition is often discussed in media reports as a potential contributor to online shaming [see 15], as well as within related literature [e.g., 19]. Online disinhibition also overlaps with the notions of anonymity presented within the qualitative findings. In the regression models, social vigilantism was one of the largest unique predictors of online shaming intentions, and also accounted for unique variance in perceived deservedness. As for online disinhibition, it also accounted for unique variance in intentions to online shame, but not perceived deservedness.

### Personality predictors: Machiavellianism, narcissism, and psychopathy

Machiavellianism had moderate positive correlations with both online shaming subscales, narcissism had small positive associations with both subscales, and psychopathy had a moderate to large positive relationship with online shaming intentions and a small positive relationship with perceived deservedness. This suggests participants who are more inclined to be deceitful, and exploitative to meet their goals (machiavellianism), who possess a sense of entitlement and a tendency for grandiose self-promoting (narcissism), and are characterised by a higher degree of impulsiveness, callousness, and selfishness (psychopathy), may be more likely to engage in online shaming and believe people to be deserving of online shaming. This also aligns with previous related research [e.g., 21] and has similarities to some notions described within reports (e.g., links to schadenfreude) in the media [15]. In the regression models, psychopathy was the largest unique predictor of intentions to online shame. However, no other personality predictor accounted for unique variance in either subscale, indicating psychopathic tendencies appear to be the most important dark personality trait assessed here for predicting online shaming.

### Strengths, limitations, and future research

As the current paper is the first known study to empirically examine several currently untested predictors of online shaming, this research is strengthened by its originality. Considering empirical research in this domain is currently limited, another key strength was the inclusion of an array of potential predictors from various domains, and this study is also bolstered by the completion of pilot testing prior to data collection taking place. Given research [45] suggests effect sizes within social psychology research are typically small to medium (by Cohen’s [38] conventions,) the medium and large effect sizes reported in this study make a worthwhile contribution to understanding why online shaming occurs. Other notable strengths include statistically controlling for social desirability, as well as advertising the study as ‘online engagement’ rather than ‘online shaming’ to minimise potential biases that may accompany the idea of shaming others. Sampling was not bound by any geographical restrictions (mirroring the globally accessible nature of the internet), which enhances the ecological validity of the current study. However, it should be noted that the current sample was skewed towards younger, Caucasian Australian university students who were employed and women. Future studies should endeavour to recruit more representative samples to gather a more generalisable understanding of online shaming engagement.

Given that, anecdotally, there are many differing understandings of what online shaming is, this study is also strengthened by the decision to provide participants with a definition of online shaming before completing questions relating to online shaming. However, given a specific shaming scenario was not used it is likely participants were envisioning different examples of online shaming when responding, in line with the availability heuristic [46]. Future exploration comparing different types of specific online shaming scenarios would be beneficial (e.g., featuring vignettes of different online shaming victims or varying social norm violations; or perhaps comparing online shaming across different social media platforms). The notion within the qualitative findings that people are held to differing standards when it comes to online shaming (e.g., being willing to shame public figures online but not everyday people) also substantiates this need to explore what conditions need to be met for people to be willing to engage in online shaming. It should also be noted that the moral choices individuals make in real life tend to be more self-serving than what hypothetical choices in research might capture [see 47]. Likewise, there is ongoing debate surrounding whether it is best to capture participant behaviours through self-reported intentions or via experimental means, as the use of hypothetical scenarios has historically been criticised as an inaccurate way to measure behaviour [48]. Given this, future research using experimental methodologies (i.e., simulated online shaming scenarios where participants can respond as if the posts were real) may produce more accurate reflections of online shaming participation.

Although the qualitative strand of this study provides some interesting and novel findings that could not be captured by the quantitative strand alone, the use of a short open-ended question does not allow for any clarification of participant meanings, and the depth of responses is limited. For instance, while participants mentioned that online shaming had destructive effects, there was no opportunity to uncover what exactly these consequences were perceived to be. Additionally, it should be noted that not all participants in this study completed the open-ended question, meaning some self-selection bias may be present in the qualitative dataset (e.g., some participants who chose to take the additional time to provide a qualitative response may have done so due to feeling more strongly about certain views, having personal experience with online shaming, etc.). Semi-structured interviews exploring a range of perceptions and experiences of online shaming would allow for a deeper account of the complexities of this phenomenon.

### Implications and conclusions

A key implication arising from this study is that the findings substantiate many previously posed but untested theoretical and media-driven explanations for online shaming, demonstrating the importance of multiple different factors in understanding this phenomenon. This provides academic and public discourse surrounding online shaming with a much-needed empirical basis and shift away from the current overreliance on purely theoretical and anecdotal speculation. This paper also offers a new validated measure to capture online shaming engagement, with two subscales which demonstrate that certain underlying mechanisms appear to be differentially responsible for a) individuals believing people to be deserving of being shamed online, and b) being willing to actually conduct online shaming.

As for practical implications, having an evidence-based understanding of what drives online shaming is an essential first step for policy makers to appropriately inform legislation. Additionally, when establishing formal guidelines and educational campaigns, the predictors tested in this study should be taken into consideration. For example, interventions could be designed (e.g., by social media companies themselves) to encourage empathy for others online and remind users to think before posting and that the individual being shamed is in fact a real person. While psychologists and other health professionals also need to have a comprehensive understanding when working with victims of online shaming, these findings may also be utilised to develop strategies to assist people and companies wanting to avoid being subjected to online shaming in the future, as well as provide the broader public a better understanding of why so many of us participate in online shaming ourselves.

Combined, this mixed-methods paper offers an original and significant contribution to the currently limited online shaming literature. This study has provided empirical support for several previously untested psychological explanations of shaming engagement in digital spaces, as well as qualitative insights into public opinions surrounding potential origins, concerns, and various other perceptual nuances of this phenomenon. Whilst the current findings can be utilised to inform public understandings, policy makers, educators, and health professionals, considerable further investigation into this research domain is still needed.

## Supporting information

S1 Appendix Additional information for the development of the Online Shaming Scale. (DOCX)

S2 Appendix Normality plots for all scale measures. (DOCX)

S3 Appendix Linear and Ridge regressions using R. (DOCX)
