# Supplementary material for: Examining the role of moral, emotional, behavioural, and personality factors in predicting online shaming
Source: PLoS One. 2023 Mar 23;18(3):e0279750. doi: 10.1371/journal.pone.0279750 (PMC10035748; doi:10.1371/journal.pone.0279750)
Supplement: S1 Appendix — (DOCX) [file pone.0279750.s001.docx]

**S1 Appendix**

**Additional information for the development of the Online Shaming Scale**

This document features all measure items and additional results for the factor analyses when developing and validating the Online Shaming Scale.

***Online Shaming Scale introductory statement and original 12 items***

The following questions refer to online behaviours performed against someone as an act of shaming using Internet technologies. To be classified as online shaming, the action must have taken place because of a perceived (based on the person’s opinion) or real breaking of a social norm. A social norm is what individuals within a community believe is normal or acceptable within a group. Please indicate your agreeance with the following items in relation to online shaming.

If someone posts something inappropriate or breaks a social norm online:

1. They deserve what is coming for them.

2. I would not post about the person online as a way of embarrassing them for what they did.

3. I would show my support for punishing that person by commenting on posts shaming that person.

4. I would comment directly on the person’s post to show my disapproval.

5. I would not click “like” on posts shaming that person.

6. They do not deserve to be ridiculed for it.

7. I would make mean or negative comments on that person’s photos, updates, or tags to make that person feel bad for what they did.

8. I would post about it as a way of letting others know what that person did wrong.

9. They should not have their name dragged across the Internet, even if that person makes a bad mistake.

10. I would edit a photo or create a meme making fun of them and then post it online for others to see as a way to punish that person.

11. I would not message them to insult them.

12. I would show my disapproval by sharing posts that call them out for what they did.

***Online Shaming Scale factor analyses additional results***

Table 1

*Promax rotated factor loadings (after principal axis factoring) for the 9-item Online Shaming Scale (N = 206)*

|  | Scale Item | Factor | |
| --- | --- | --- | --- |
|  |  | I | PD |
| 7. | I would make negative or mean comments on the person’s updates, photos, or tags to make them feel bad for what they did. | .84 |  |
| 3. | I would show my support for punishing that person by commenting on posts shaming that person. | .73 |  |
| 8. | I would post about it as a way of letting others know what that person did wrong. | .73 |  |
| 10. | I would create a meme or edit a photo making fun of that person and then post it online for others to see as a way to punish them. | .71 |  |
| 4. | I would comment directly on the person’s post to show my disapproval. | .62 |  |
| 12. | I would show my disapproval by sharing posts that call them out for what they did. | .53 |  |
| 6. | They do not deserve to be ridiculed for it. ^a^ |  | .89 |
| 1. | They deserve what is coming for them. |  | .56 |
| 9. | They should not have their name dragged across the internet, even if that person makes a bad mistake.^a^ |  | .44 |

*Note*. Factor loadings < .3 are not shown. Items 2 (“I would not post about the person online as a way of embarrassing them for what they did.”), 5 (“I would not click “like” on posts shaming that person.”), and 11 (“I would not message them to insult them.”) were removed due to poor/cross-loadings. I = intentions. PD = perceived deservedness. ^a^ = Item responses were reverse coded prior to analysis.

Table 2.

*Fit indices for confirmatory factor analyses for the OSS with Kline’s (2005) cut-off criteria (robust statistics)*

| Model | NFI | NNFI | CFI | RMSEA[90% CI] | S-Bχ2/df |
| --- | --- | --- | --- | --- | --- |
| Cut-off criteria | ≥.95 | ≥.90 | ≥.95 | <.08 | ≤3.0 |
| Higher order model | .90 | .91 | .94 | .08[.05, .10] | 2.18 |
| One-factor model | .82 | .82 | .86 | .11[.09, .14] | 3.51 |
| Correlated two-factor  model | .89 | .91 | .93 | .08[.05, .11] | 2.27 |
| Uncorrelated two-factor  model | .85 | .86 | .90 | .10[.07, .12] | 2.90 |

*Note.* OSS = Online Shaming Scale; NFI = normed fit index; NNFI = non-normed fit index; CFI = comparative fit index; RMSEA = root mean square error of approximation; CI = confidence interval; S-Bχ2 = Satorra-Bentler chi-squared; df = degrees of freedom.

Figure 1. Higher order confirmatory factor analysis model of the OSS.
