# Supplementary material for: Examining the role of moral, emotional, behavioural, and personality factors in predicting online shaming
Source: PLoS One. 2023 Mar 23;18(3):e0279750. doi: 10.1371/journal.pone.0279750 (PMC10035748; doi:10.1371/journal.pone.0279750)

**S2 Appendix**

**Normality plots for all scale measures**


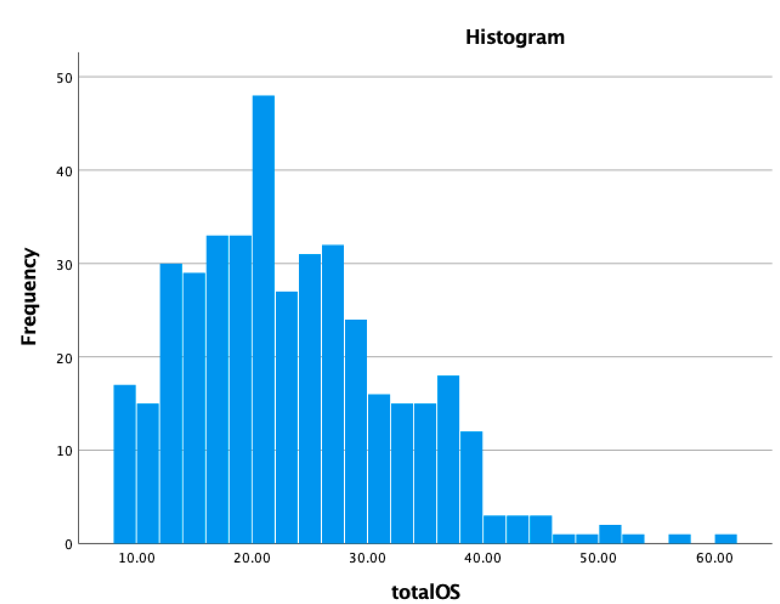
*Figure 1. Total OSS plot. Figure 2. OSS intentions subscale plot.*

**
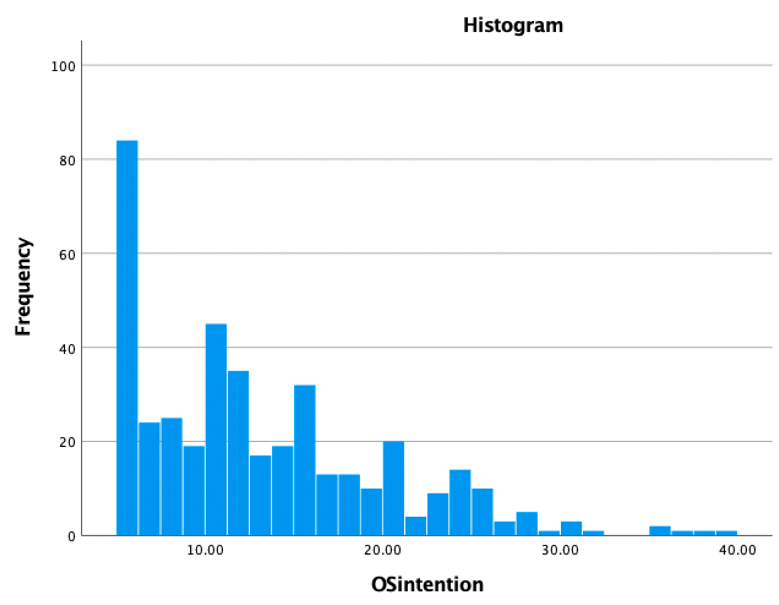
**

**
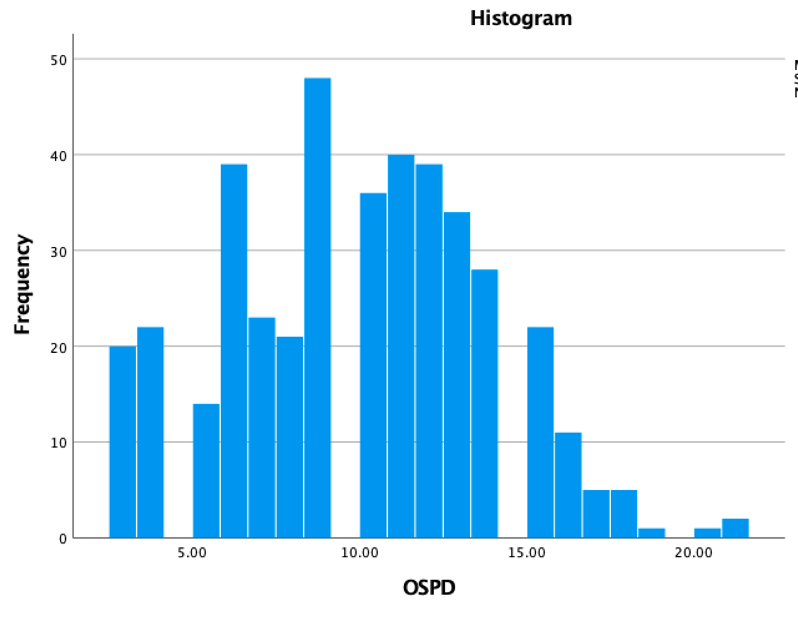
***Figure 3. OSS perceived deservedness subscale plot. Figure 4. Moral grandstanding plot.*


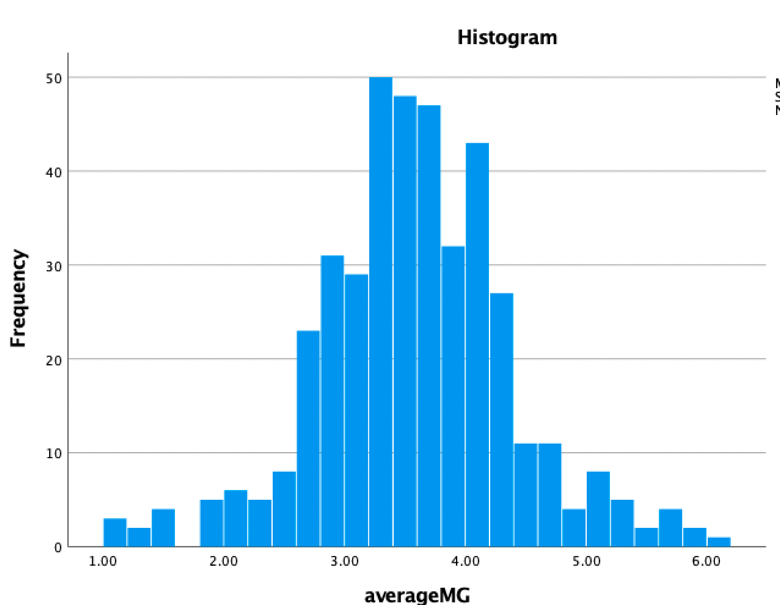


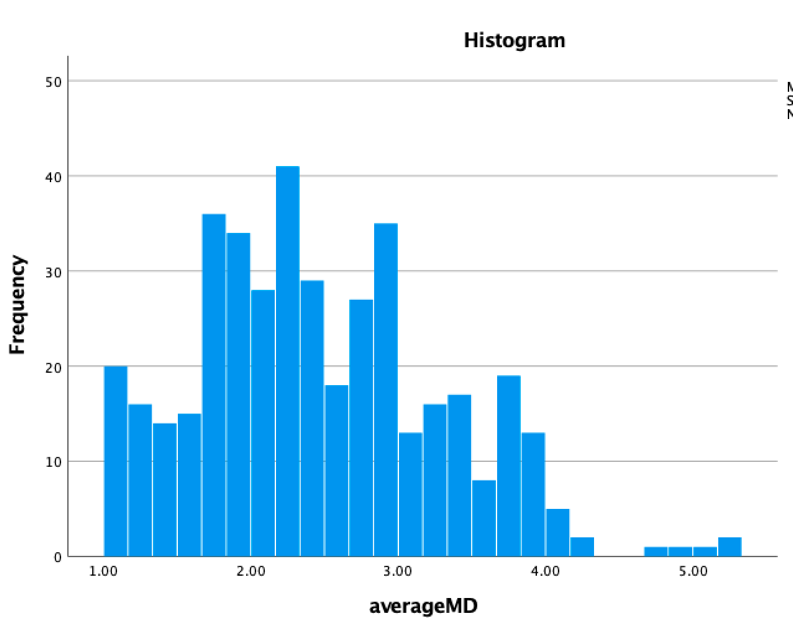
*Figure 5. Moral disengagement plot. Figure 6. Emotional reactivity plot.*


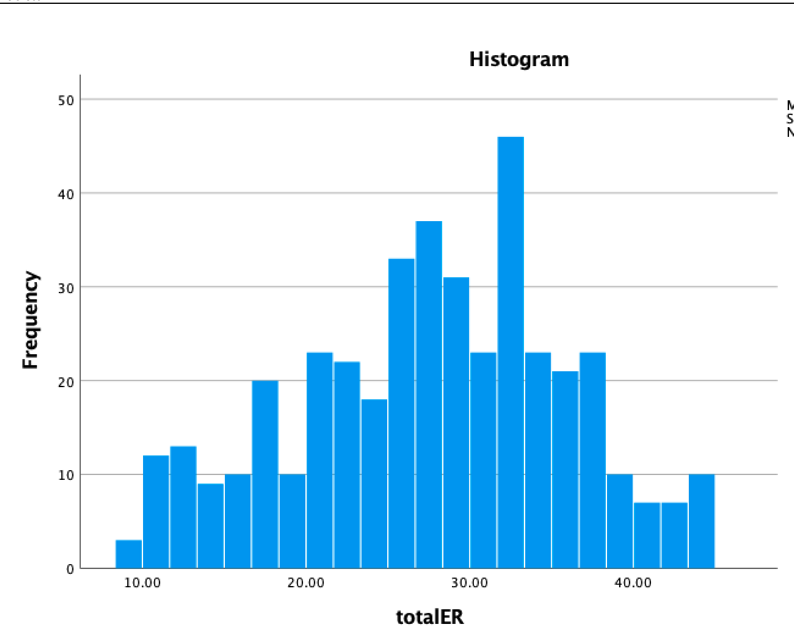


*Figure 7. Empathy plot. Figure 8. Social vigilantism plot.*


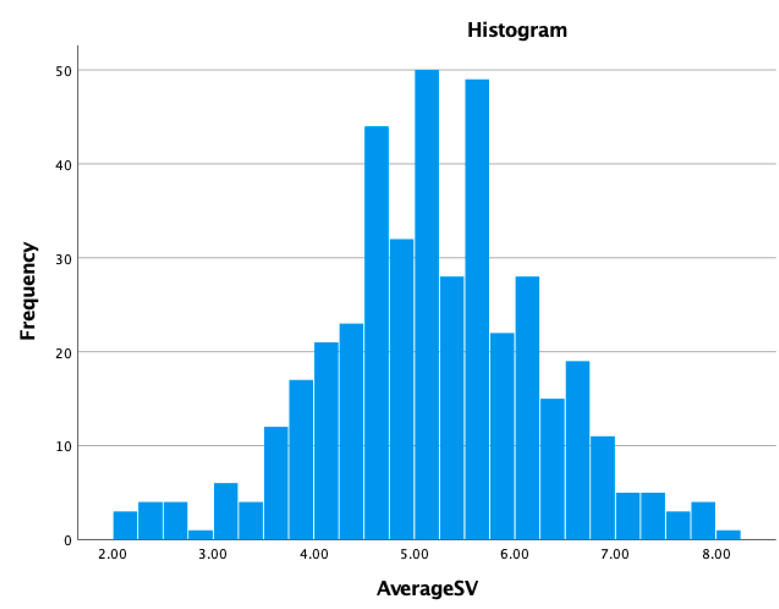

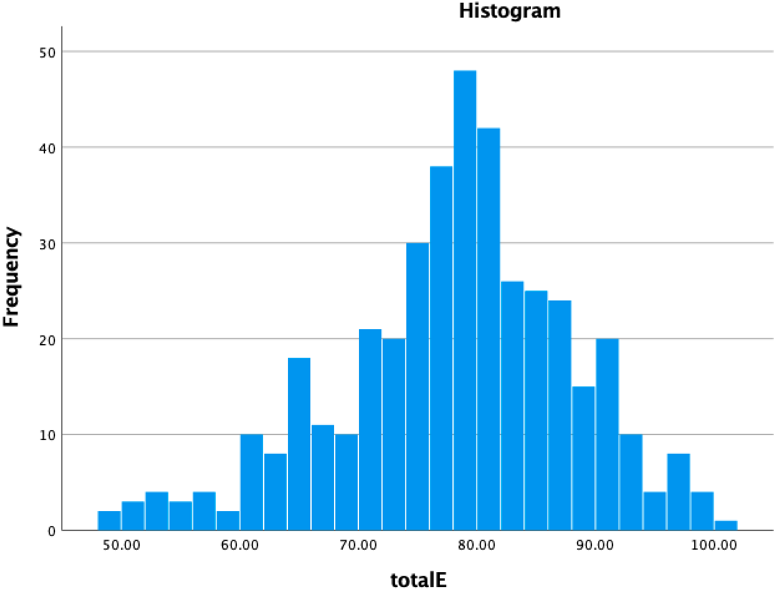


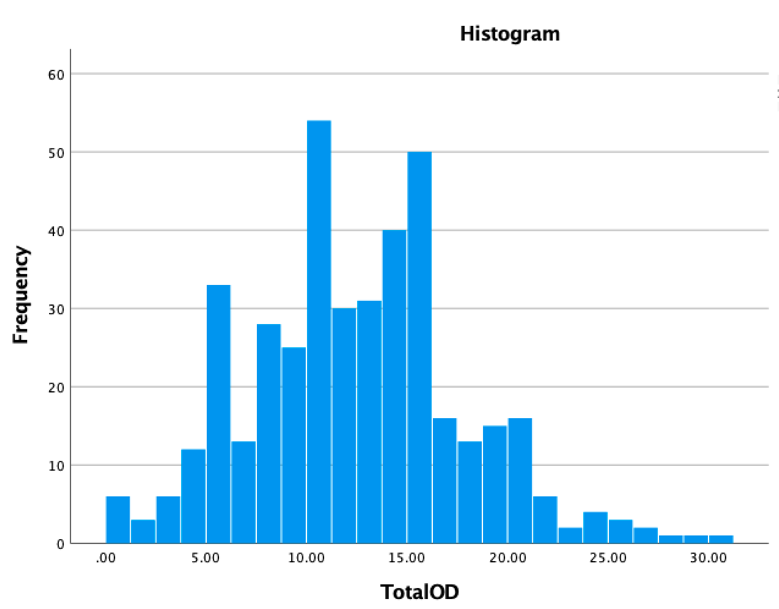

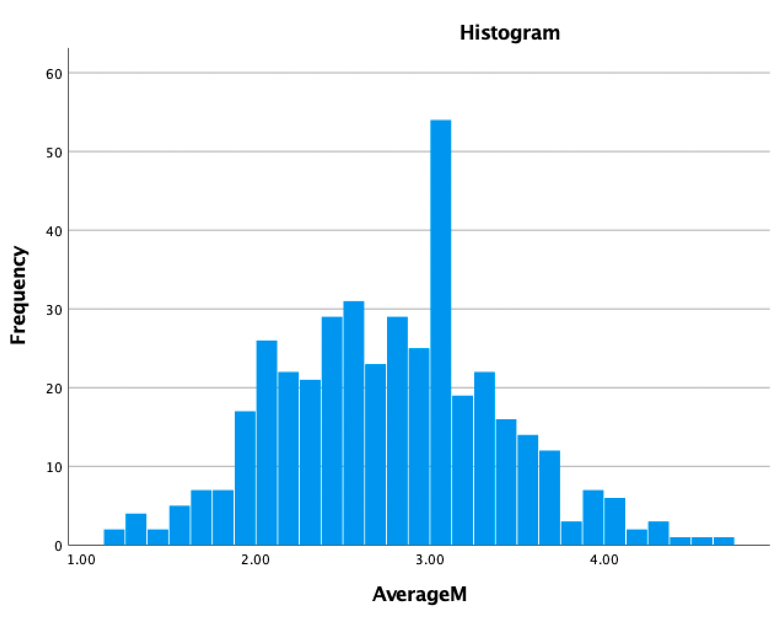
*Figure 9. Online disinhibition plot. Figure 10. Machiavellianism plot.*


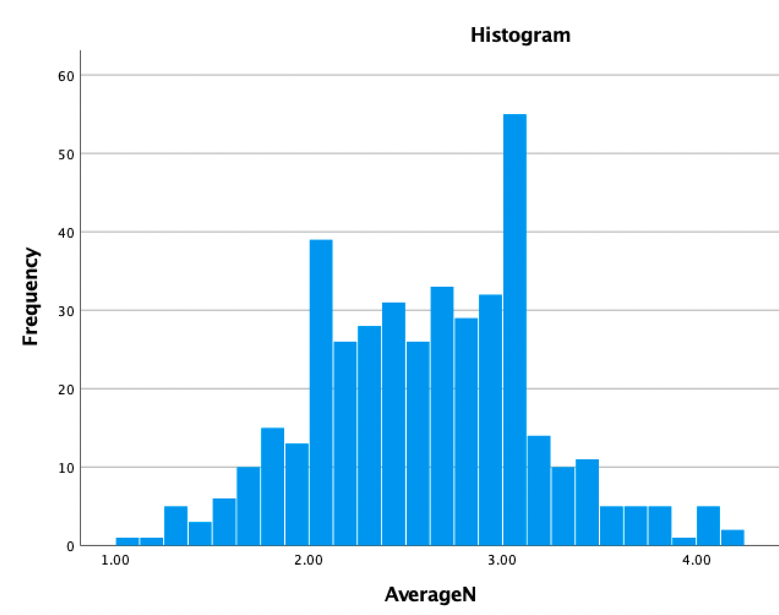
*Figure 11. Narcissism plot. Figure 12. Psychopathy plot.*


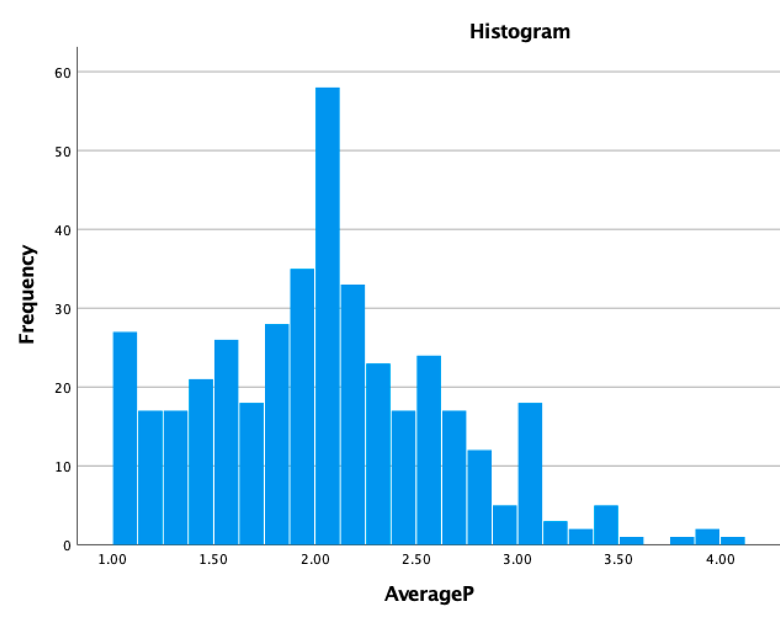


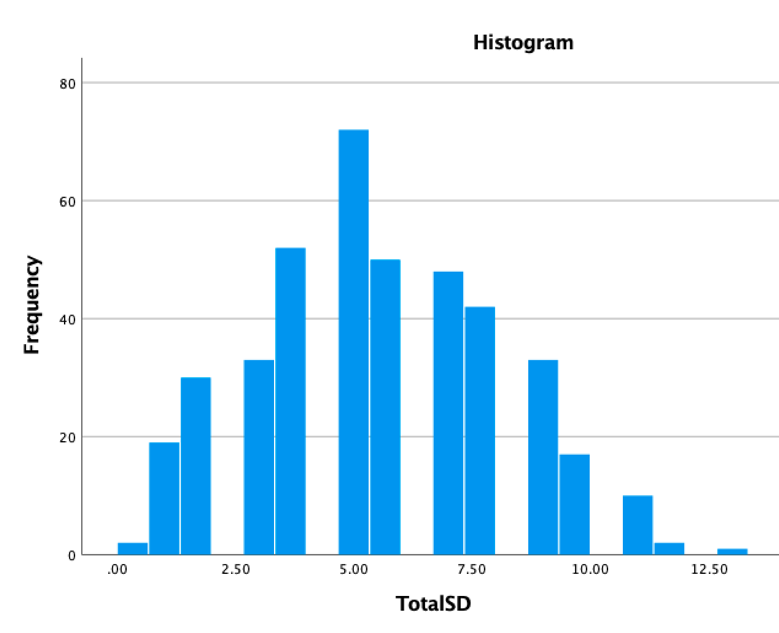
*Figure 13. Social desirability plot. Figure 14. OSS intentions plot after transformation.*


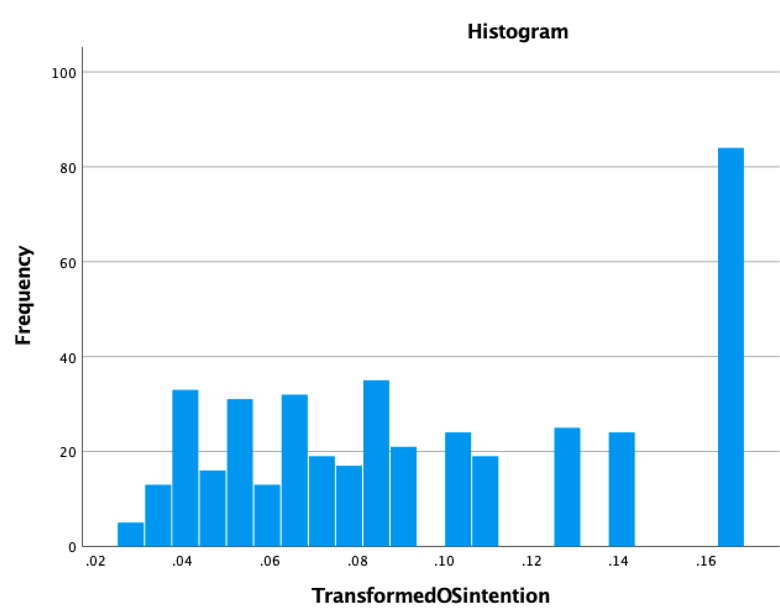

Supplement: S2 Appendix — (DOCX) [file pone.0279750.s002.docx]
