# Supplementary material for: Examining the role of moral, emotional, behavioural, and personality factors in predicting online shaming
Source: PLoS One. 2023 Mar 23;18(3):e0279750. doi: 10.1371/journal.pone.0279750 (PMC10035748; doi:10.1371/journal.pone.0279750)
Supplement: S3 Appendix — (DOCX) [file pone.0279750.s003.docx]

**S3 Appendix**

**Linear and Ridge regressions using R**

To assess the robustness of the two hierarchical multiple regression analyses (HMRAs) featured in the current findings, two post hoc Ridge regressions were also conducted using the same data (see Table 1 for a summary of relevant statistics for both regression types). Whilst the Akaike information criterion (AIC) and the Bayesian information criterion (BIC) values were lower for the Ridge regressions compared to the linear regressions (suggesting the Ridge regressions were comparatively better-fit models), the R^2^ and adjusted R^2^ (AR^2^) values were the same for both regression types when predicting the online shaming intentions subscale, and were also almost identical when predicting the online shaming perceived deservedness subscale. This consistency across both regression types regarding the proportion of variance in the criterion variables accounted by the predictor variables supports the robustness of the original findings.

The mean-squared error (MSE) traced by lambda plots (featured in the Ridge regression output below) for both Ridge regressions demonstrate that the error does decrease slightly when applying some bias to the predictors. However, since the bias needed to slightly decrease this error is small, this highlights that the linear regressions are already quite strong, and therefore supports the use of the original regressions. The log lambda-coefficients plots and the Ridge regression coefficients (also featured in the Ridge regression output below) also demonstrate the same overarching findings as the original HMRAs regarding which predictors are most important in predicting the two criterion variables. For example, in both regression types, the three most important variables in predicting online shaming intentions are a) whether the participant has already shamed someone online before, b) psychopathy, and c) moral grandstanding. This consistency in the overall pattern of predictor coefficients both with and without bias applied further supports the robustness of the current findings.

**Table 1. Summary of R^2^, adjusted R^2^, AIC, and BIC values for linear and Ridge regressions**

|  |  | Linear regressions | | | | Ridge regressions | | | | | | |
| --- | --- | --- | --- | --- | --- | --- | --- | --- | --- | --- | --- | --- |
|  |  | R^2^ | AR^2^ | AIC | BIC | |  | R^2^ | AR^2^ | AIC | BIC |  |
| OSI |  |  |  |  |  | |  |  |  |  |  |  |
|  | Step 1 | .12 | .11 | 2705 | 2729 | |  | - | - | - | - |  |
|  | Step 2 | .12 | .11 | 2707 | 2735 | |  | - | - | - | - |  |
|  | Step 3 | .39 | .37 | 2573 | 2638 | |  | .39 | .37 | -6230 | -6175 |  |
| OSPD |  |  |  |  |  | |  |  |  |  |  |  |
|  | Step 1 | .03 | .02 | 2265 | 2289 | |  | - | - | - | - |  |
|  | Step 2 | .04 | .03 | 2261 | 2289 | |  | - | - | - | - |  |
|  | Step 3 | .20 | .17 | 2205 | 2269 | |  | .19 | .16 | -681 | -626 |  |

***Predicting online shaming intentions using linear regression in R***


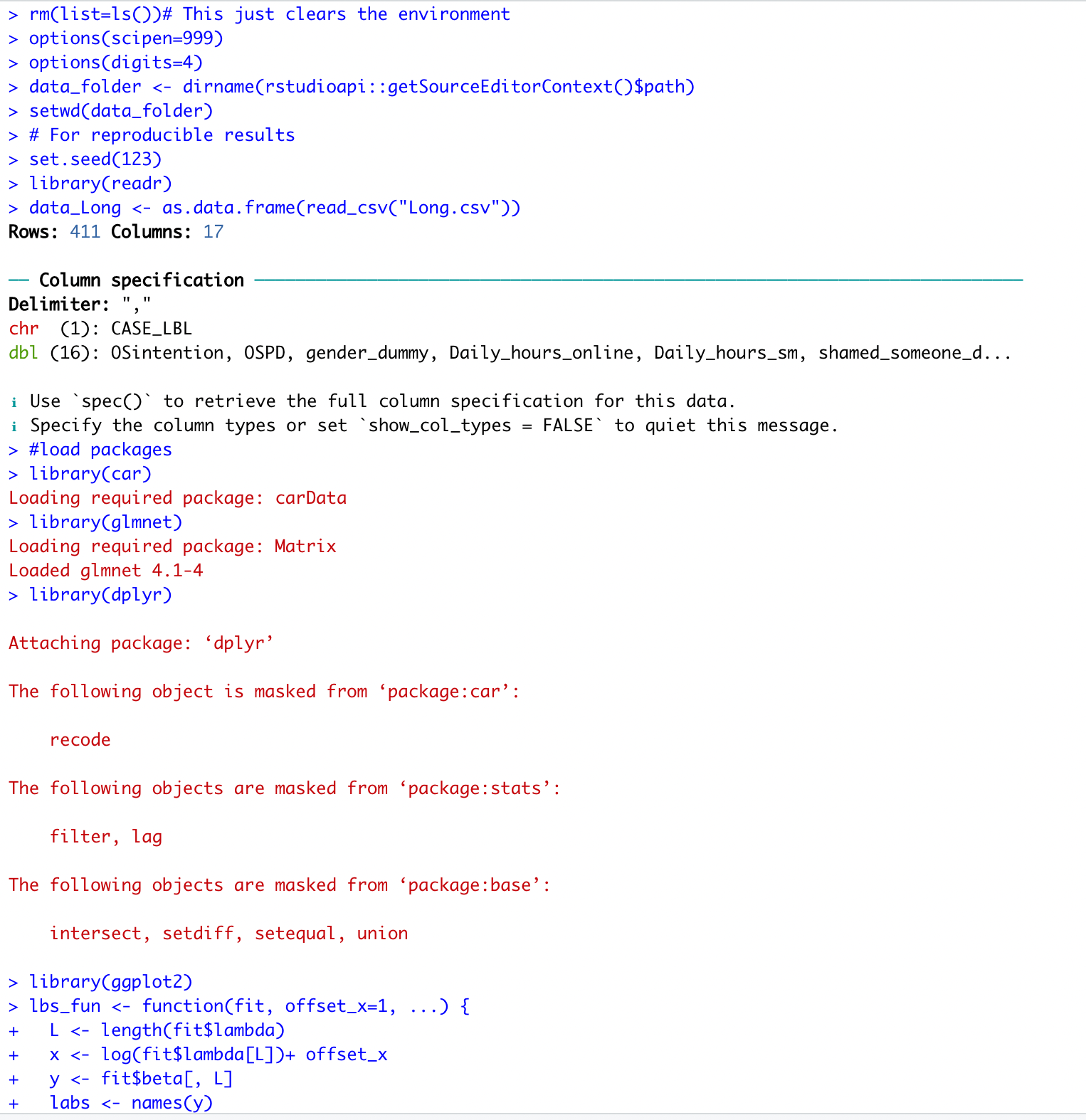


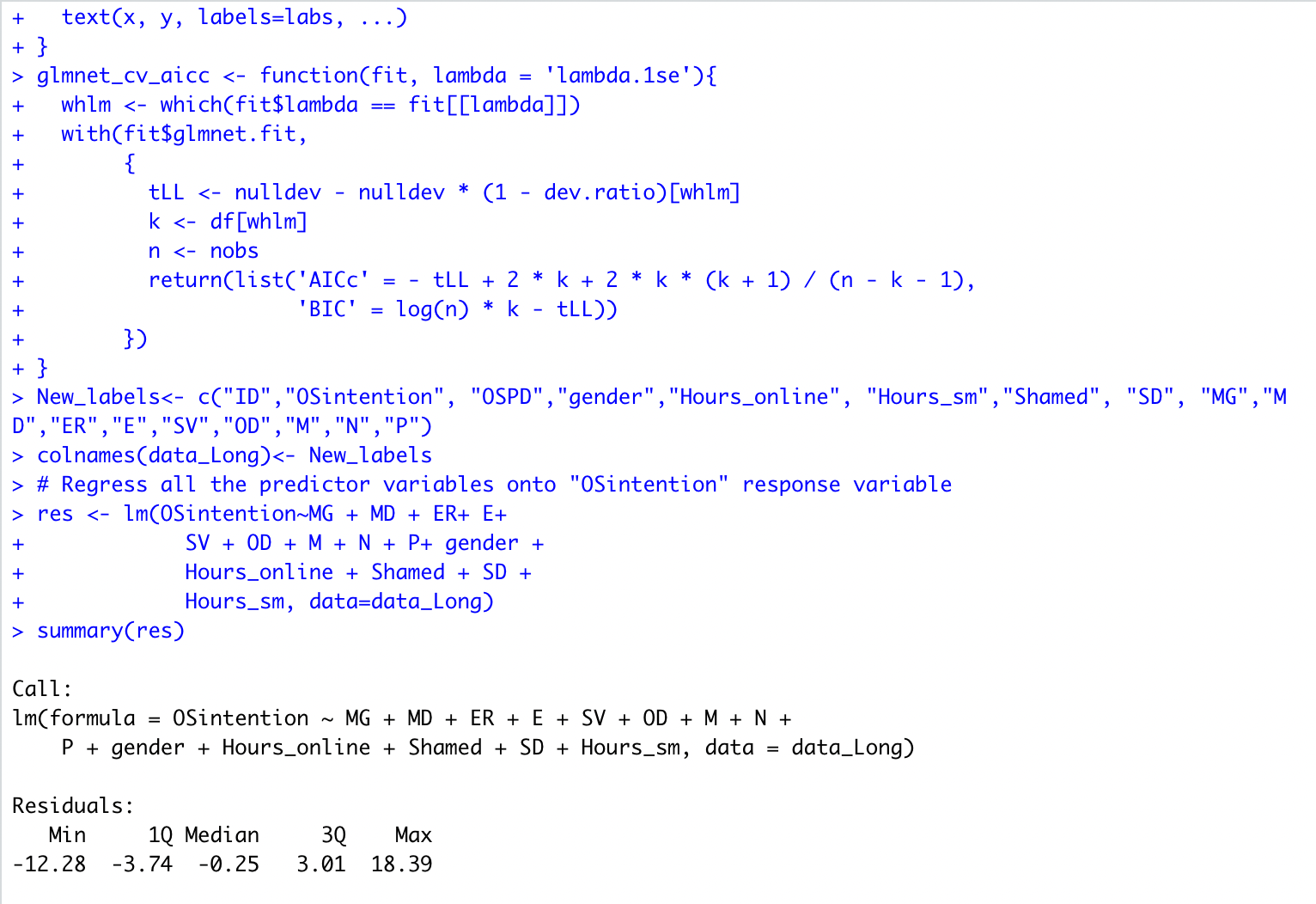


**Step 1.**

**
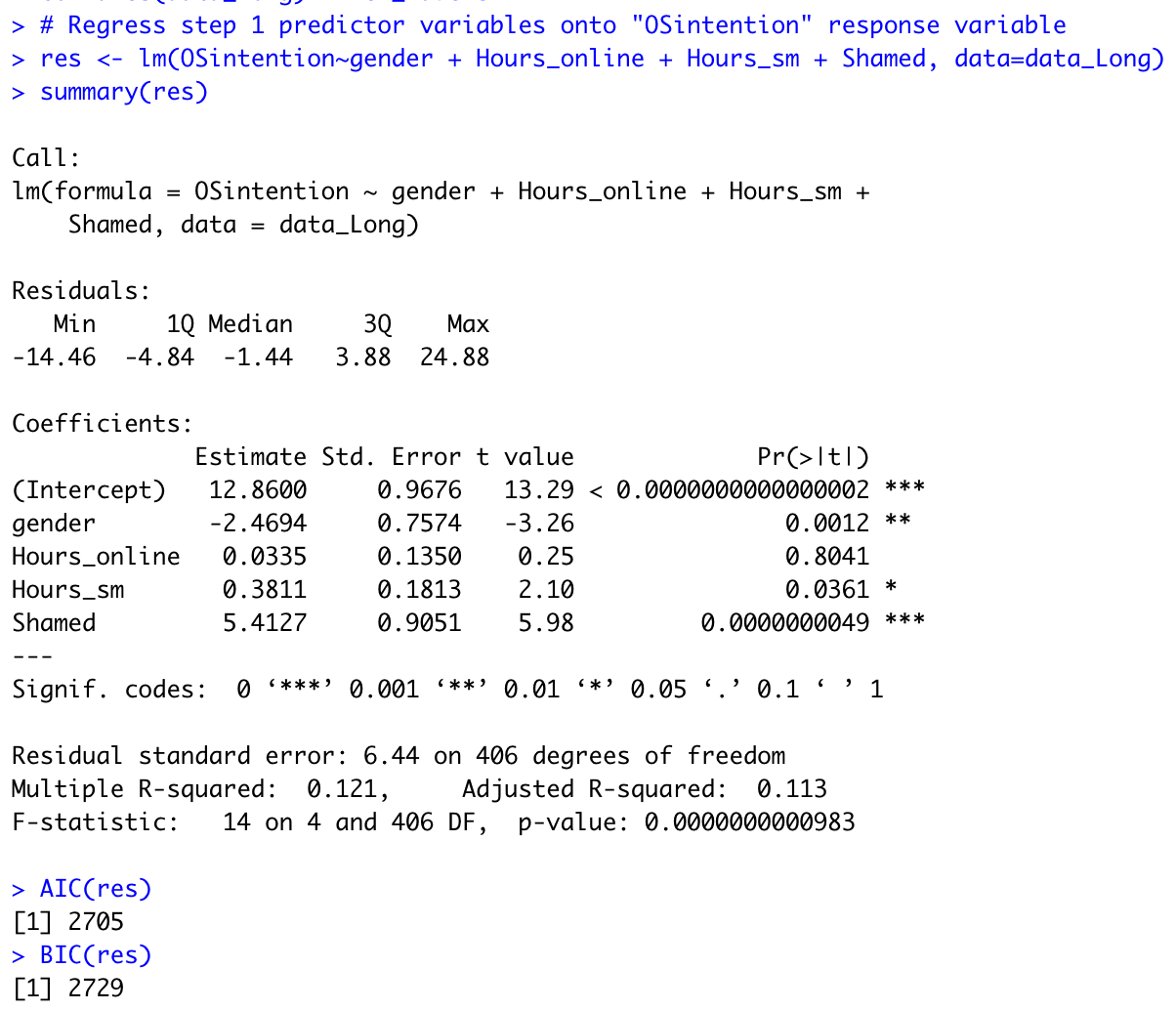
**

**Step 2.**

**
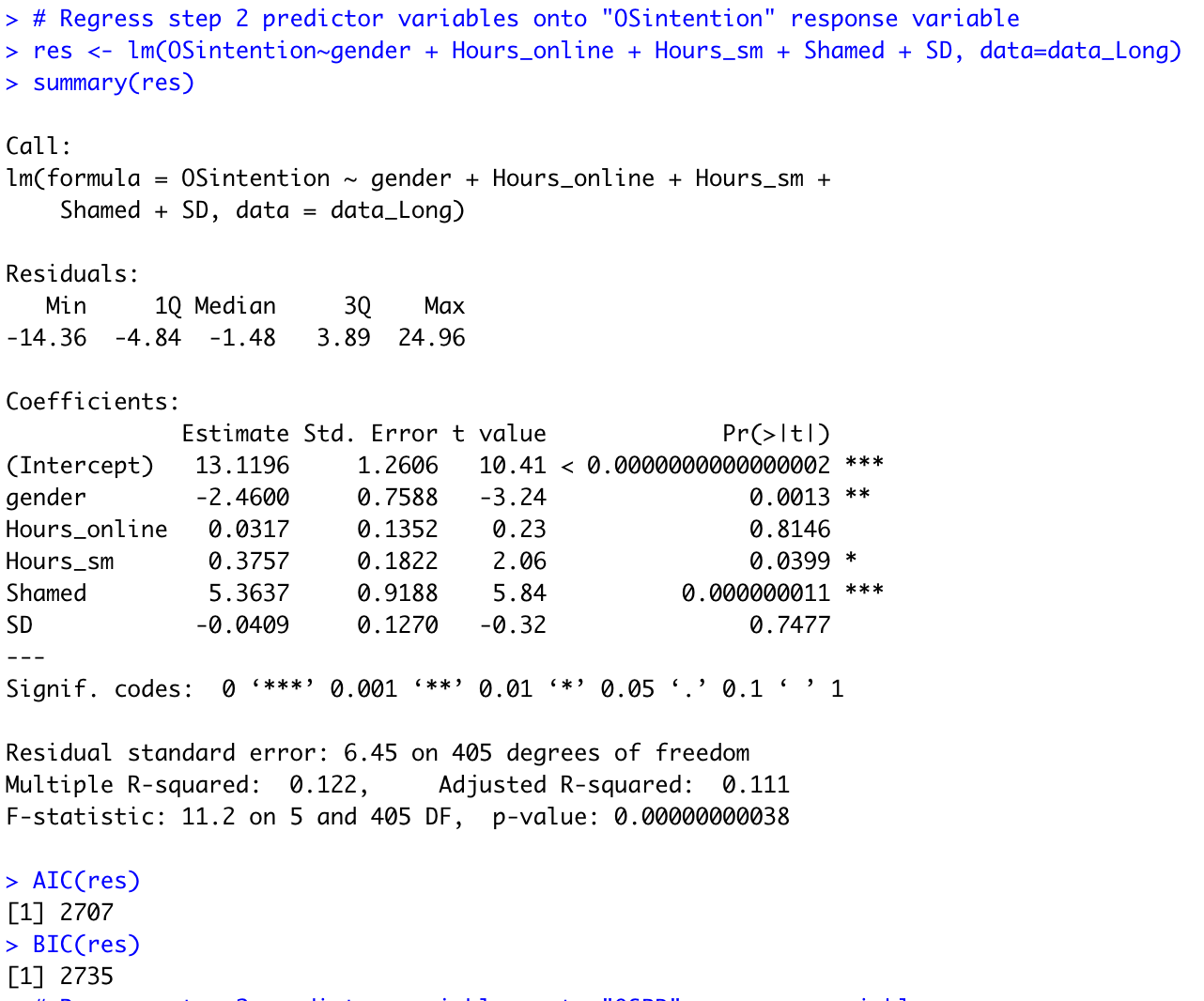
**

**Step 3.**


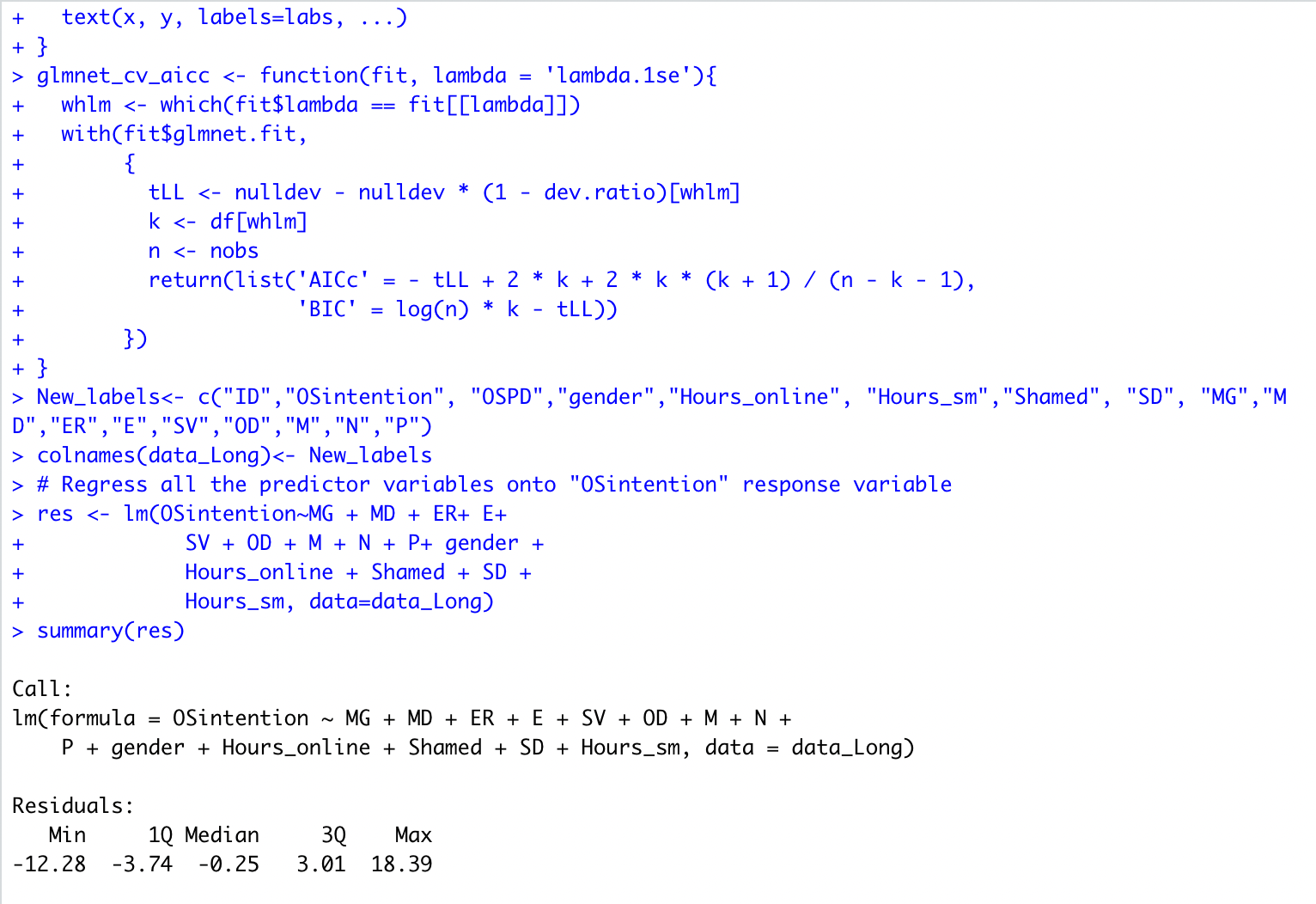


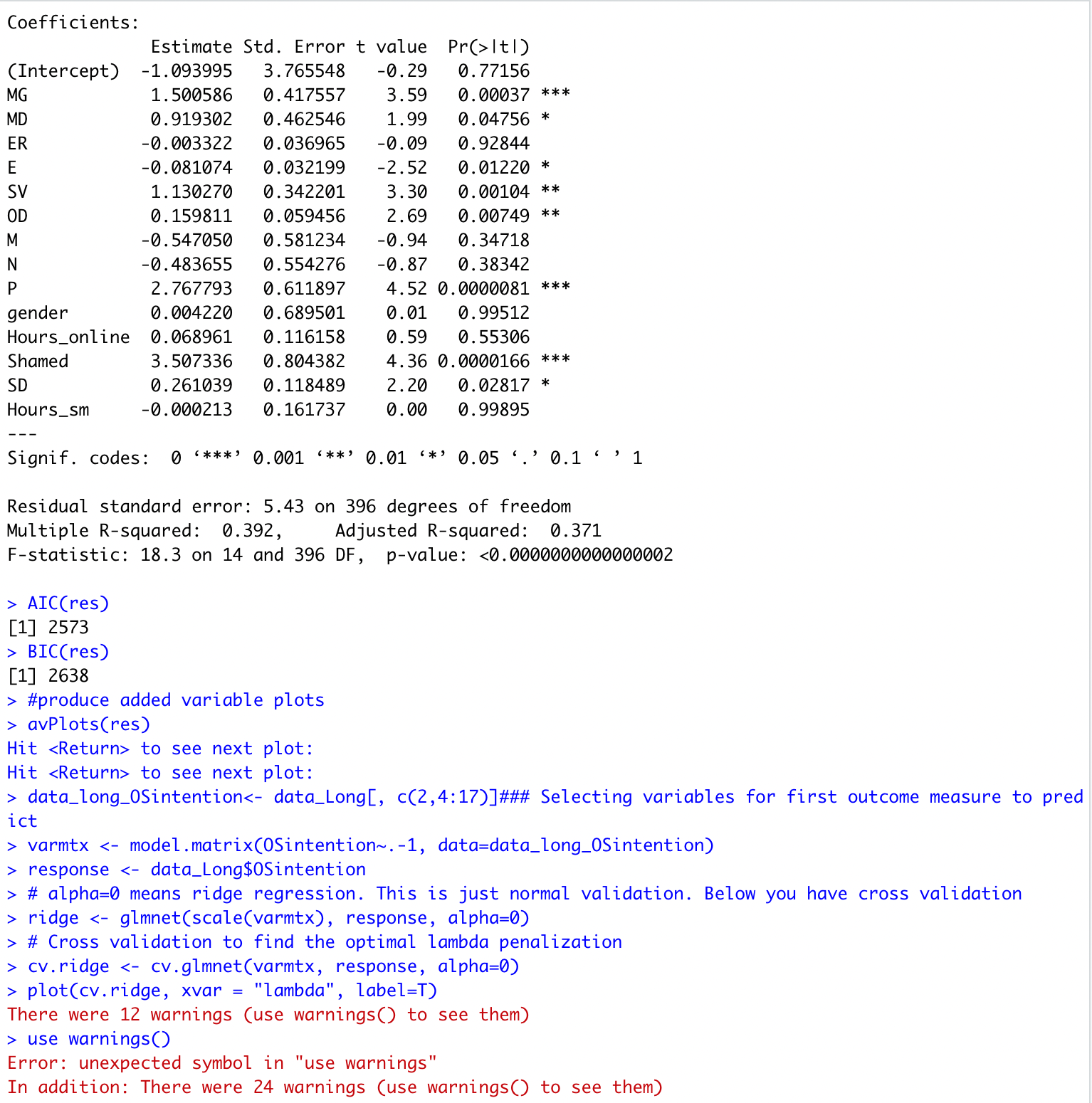


***Predicting online shaming intentions using Ridge regression in R***


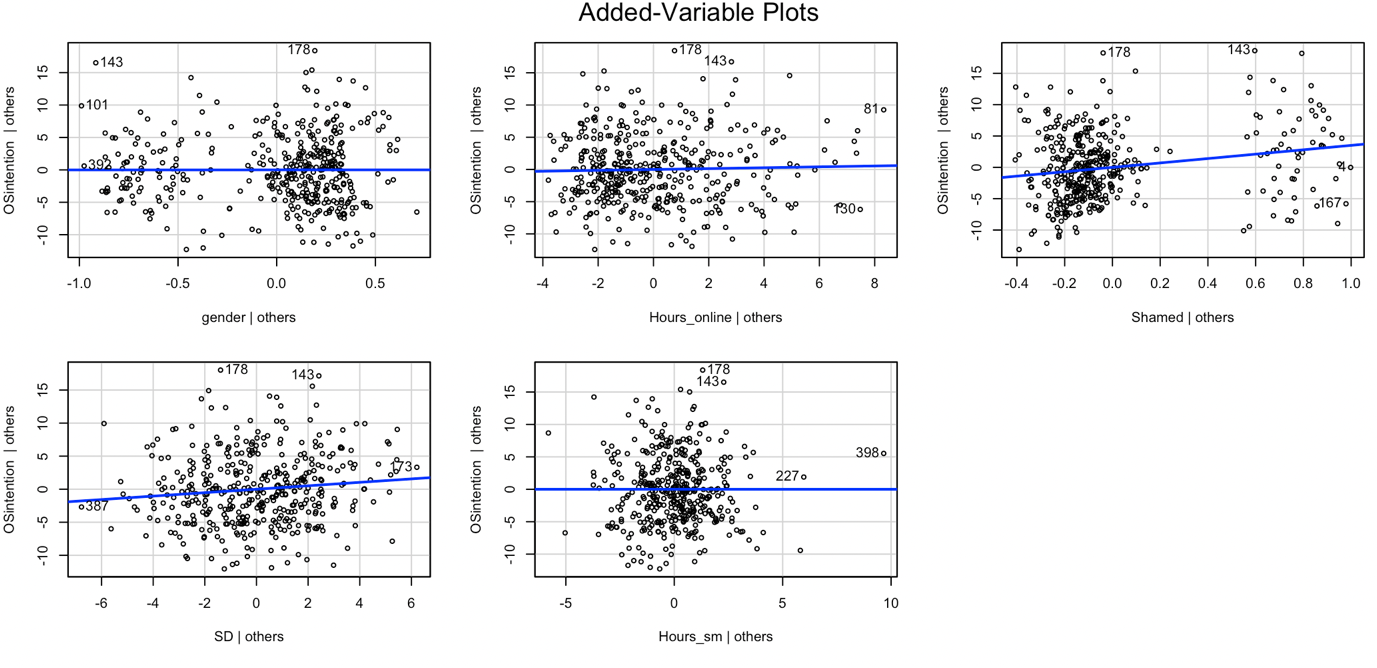


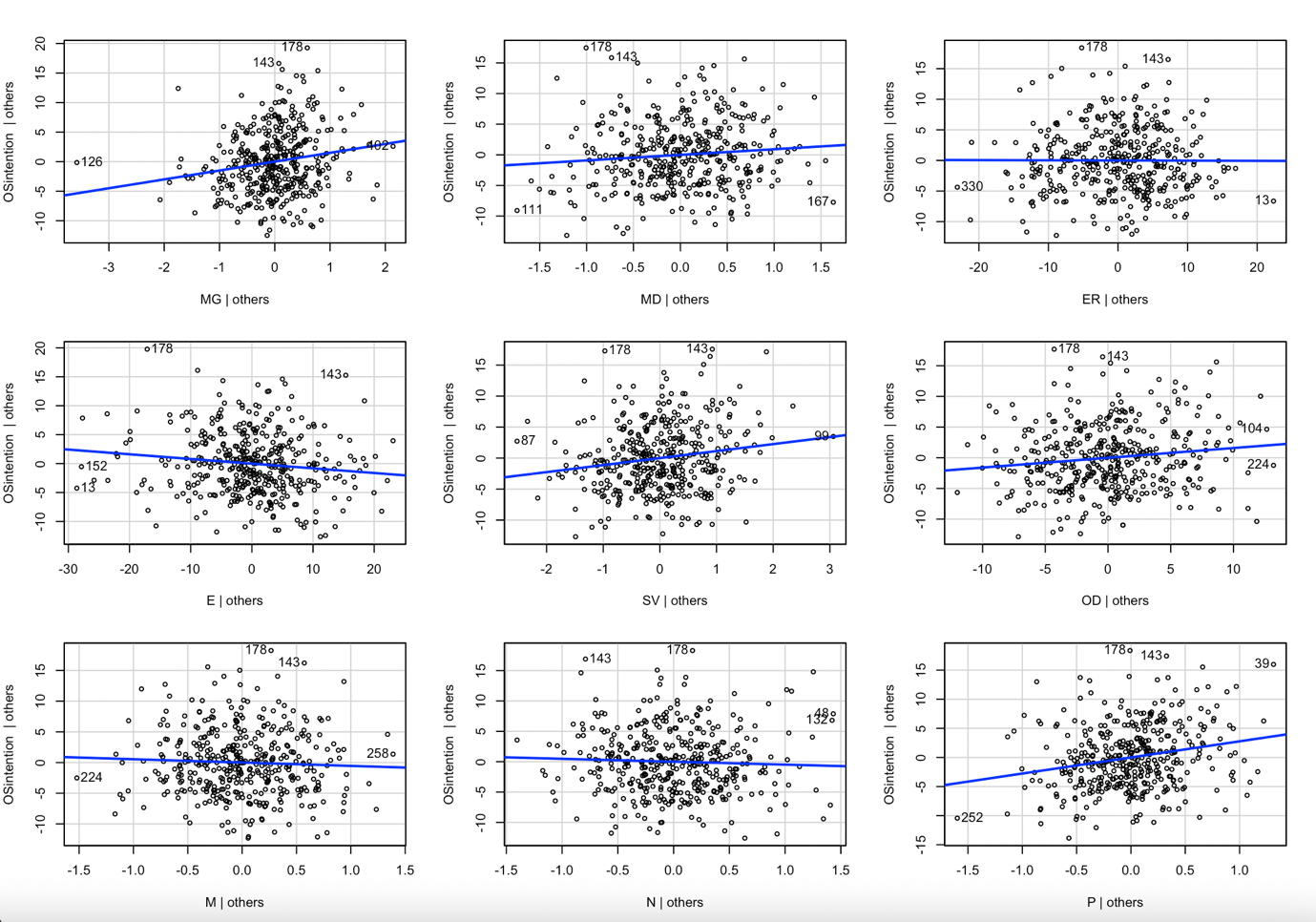


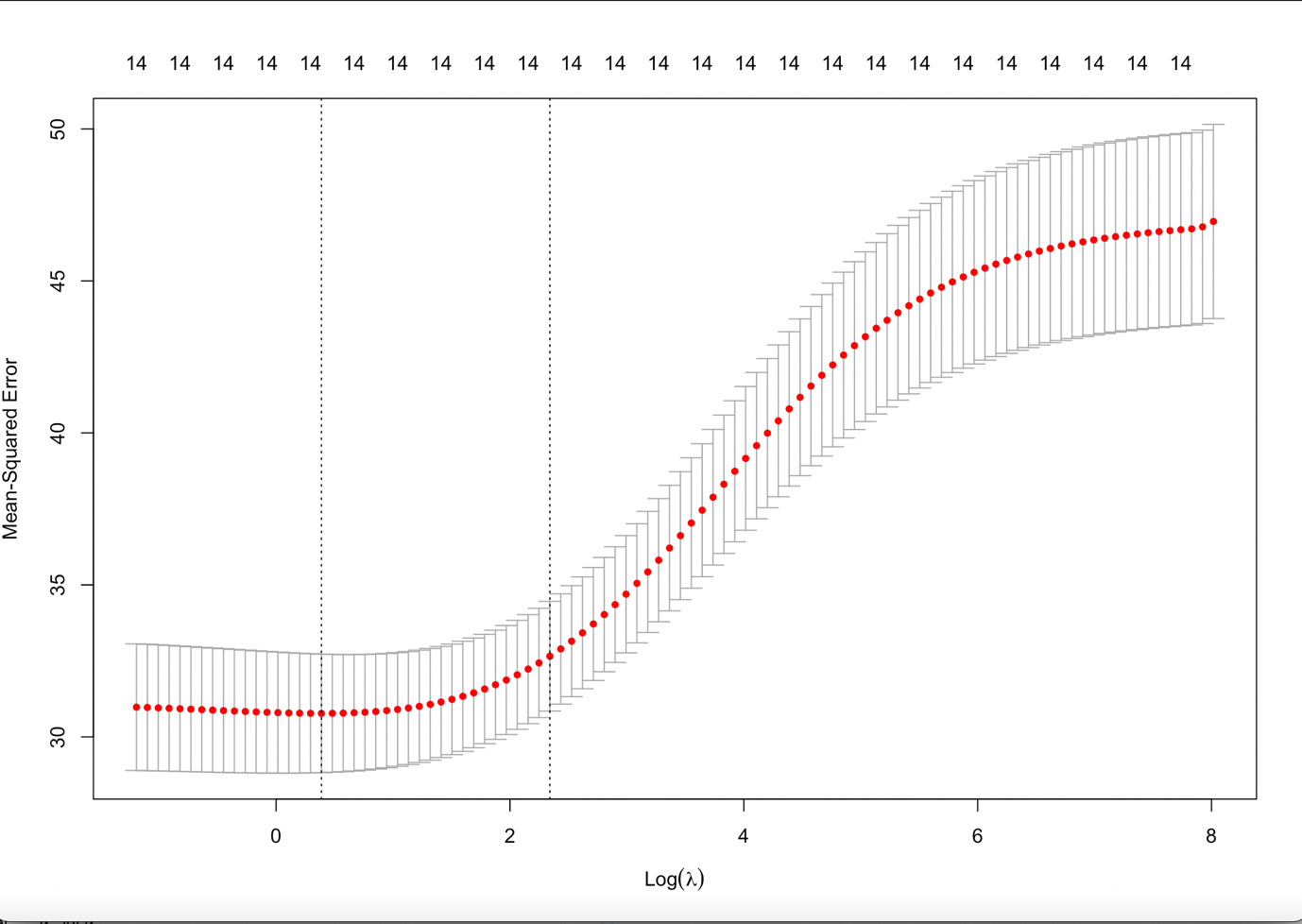


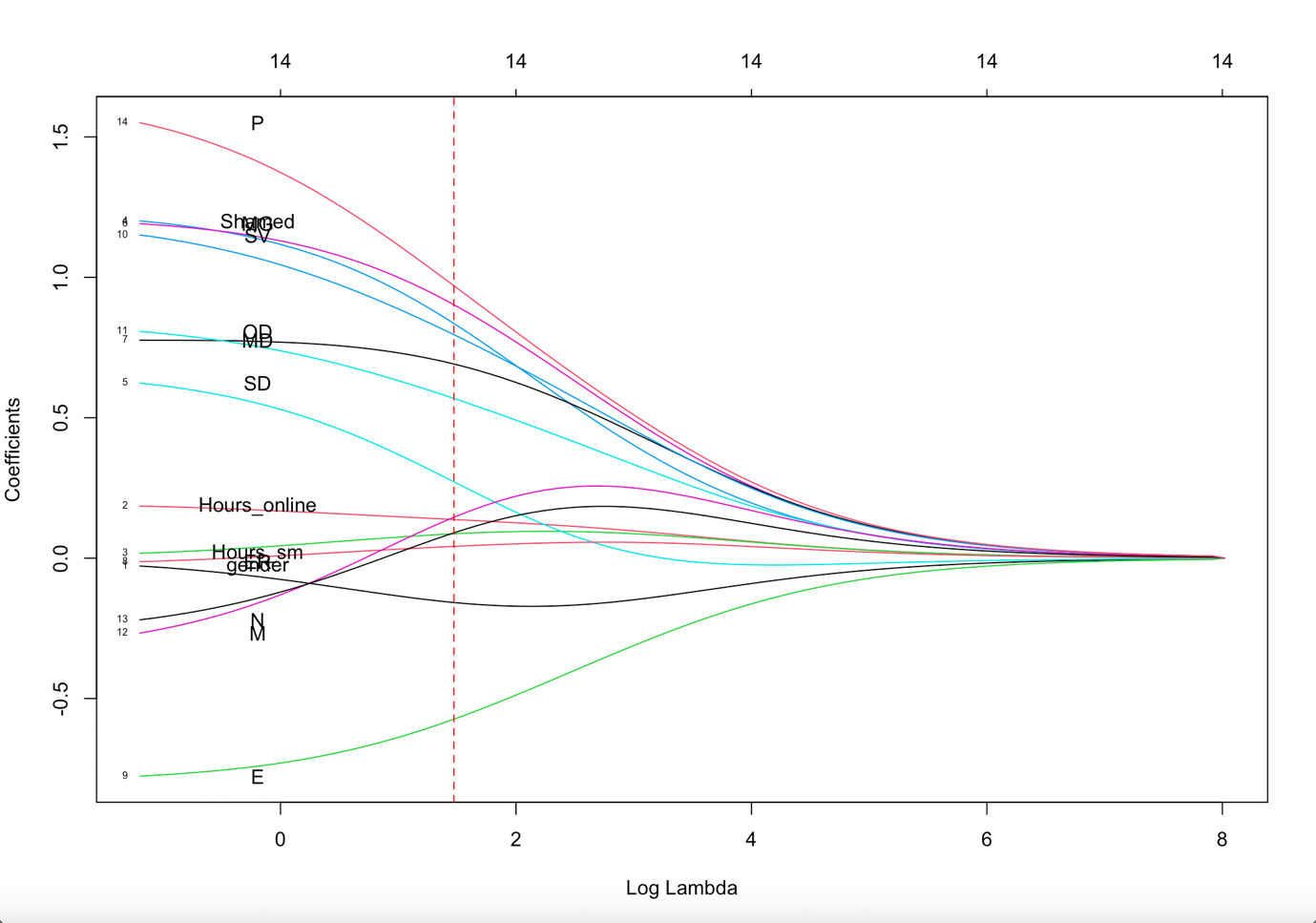


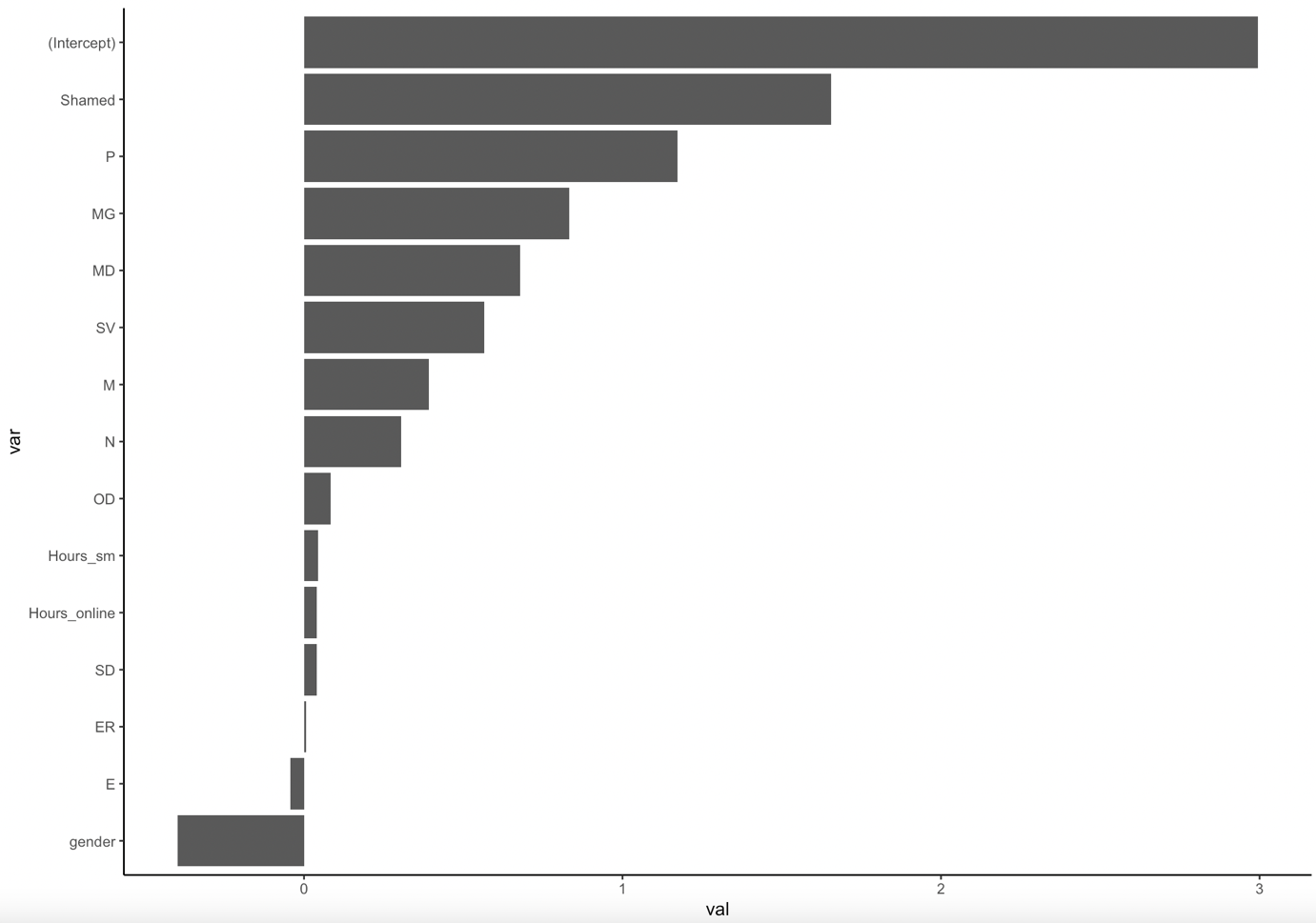


**
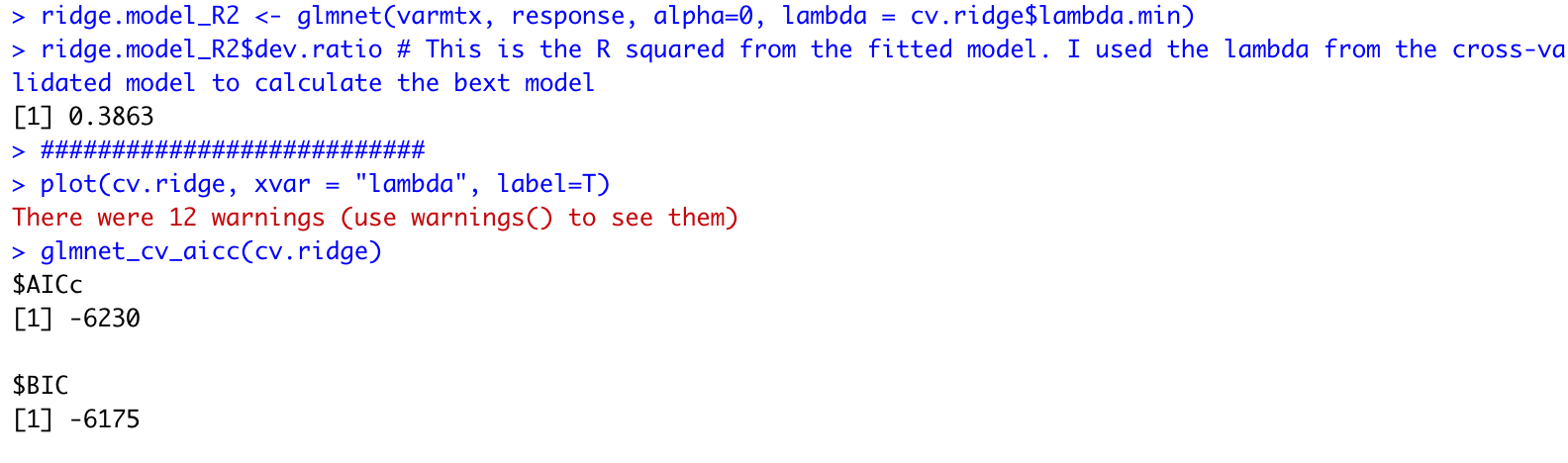
**


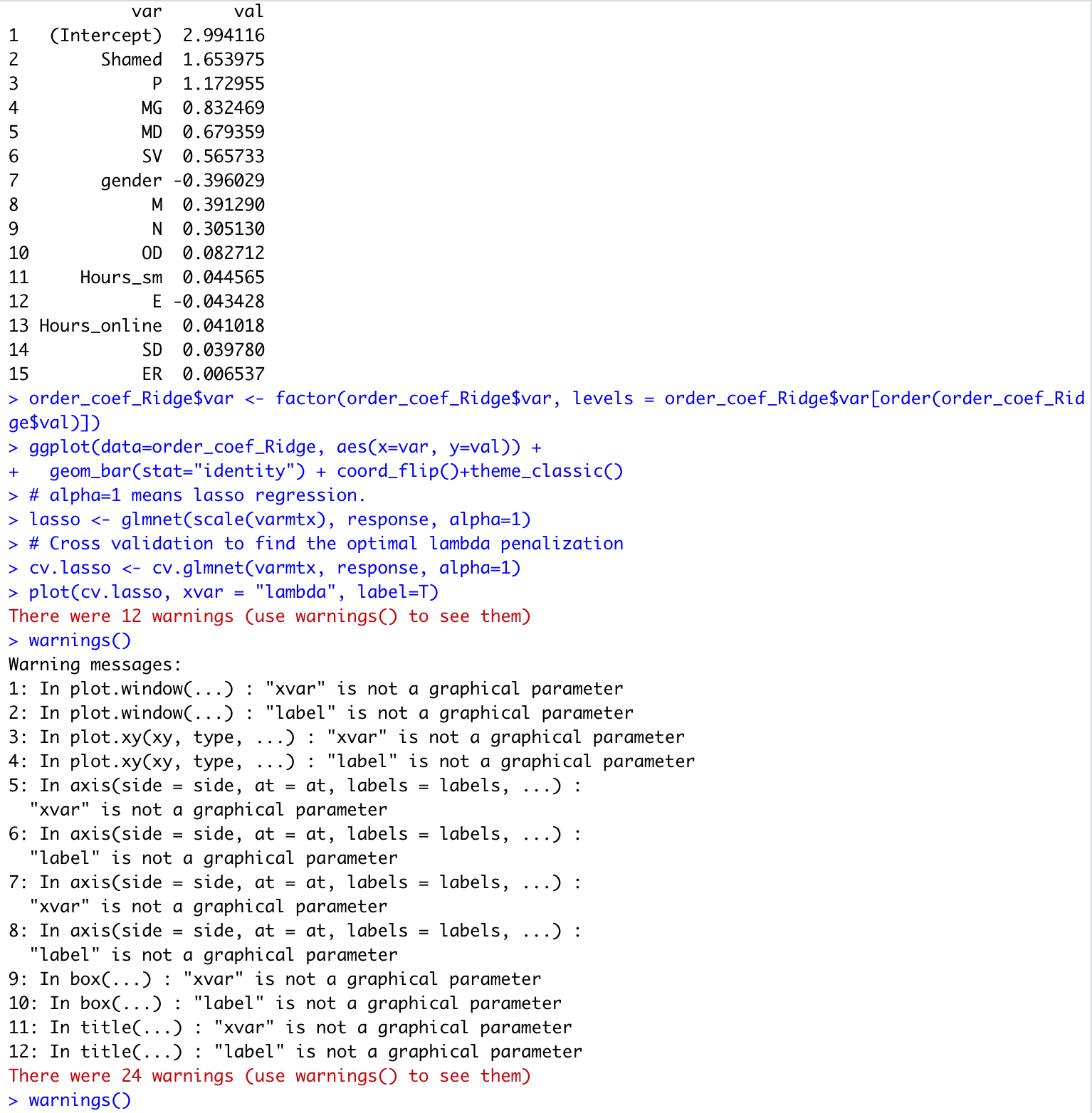


***Predicting online shaming perceived deservedness using linear regression in R***

**Step 1.**

**
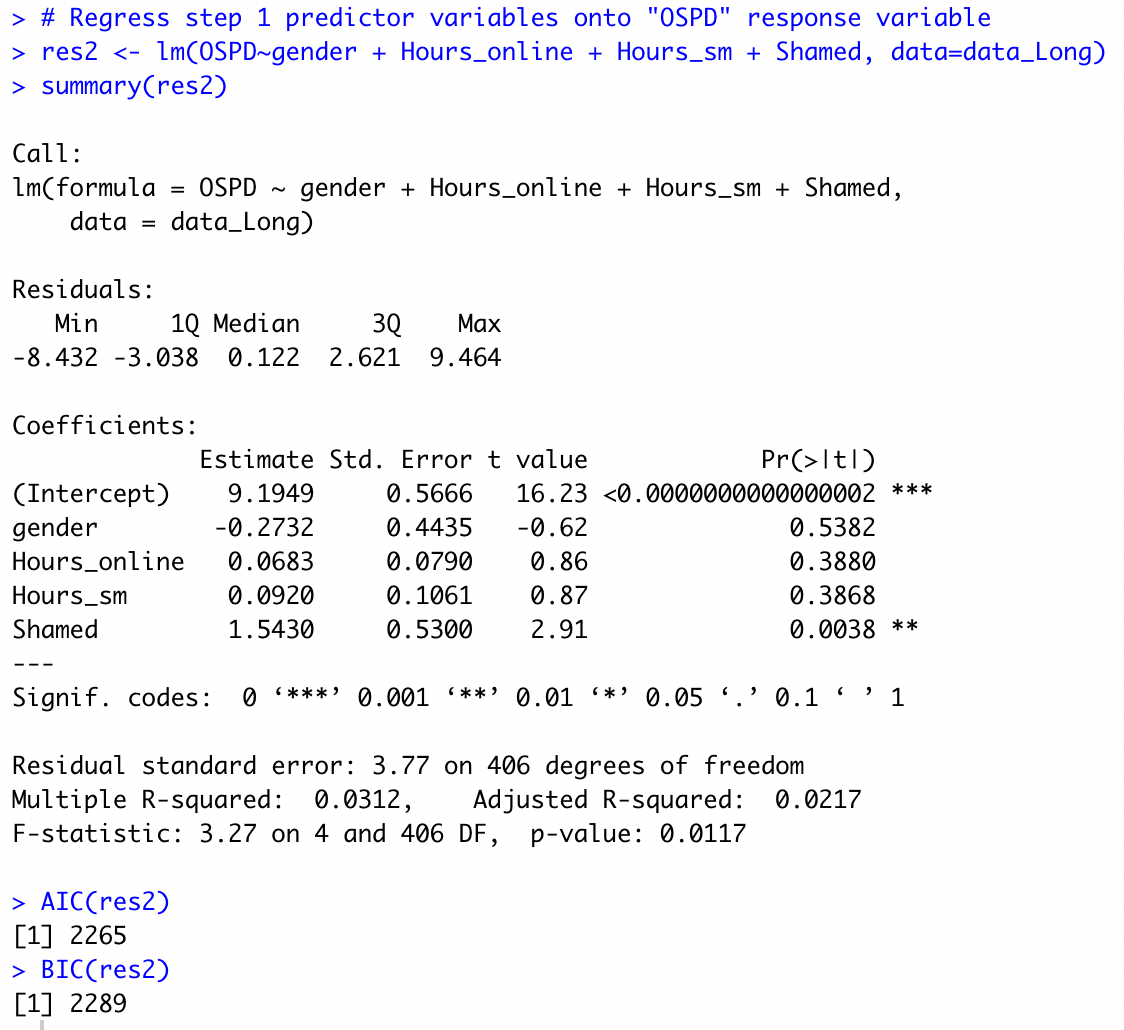
**

**Step 2.**

**
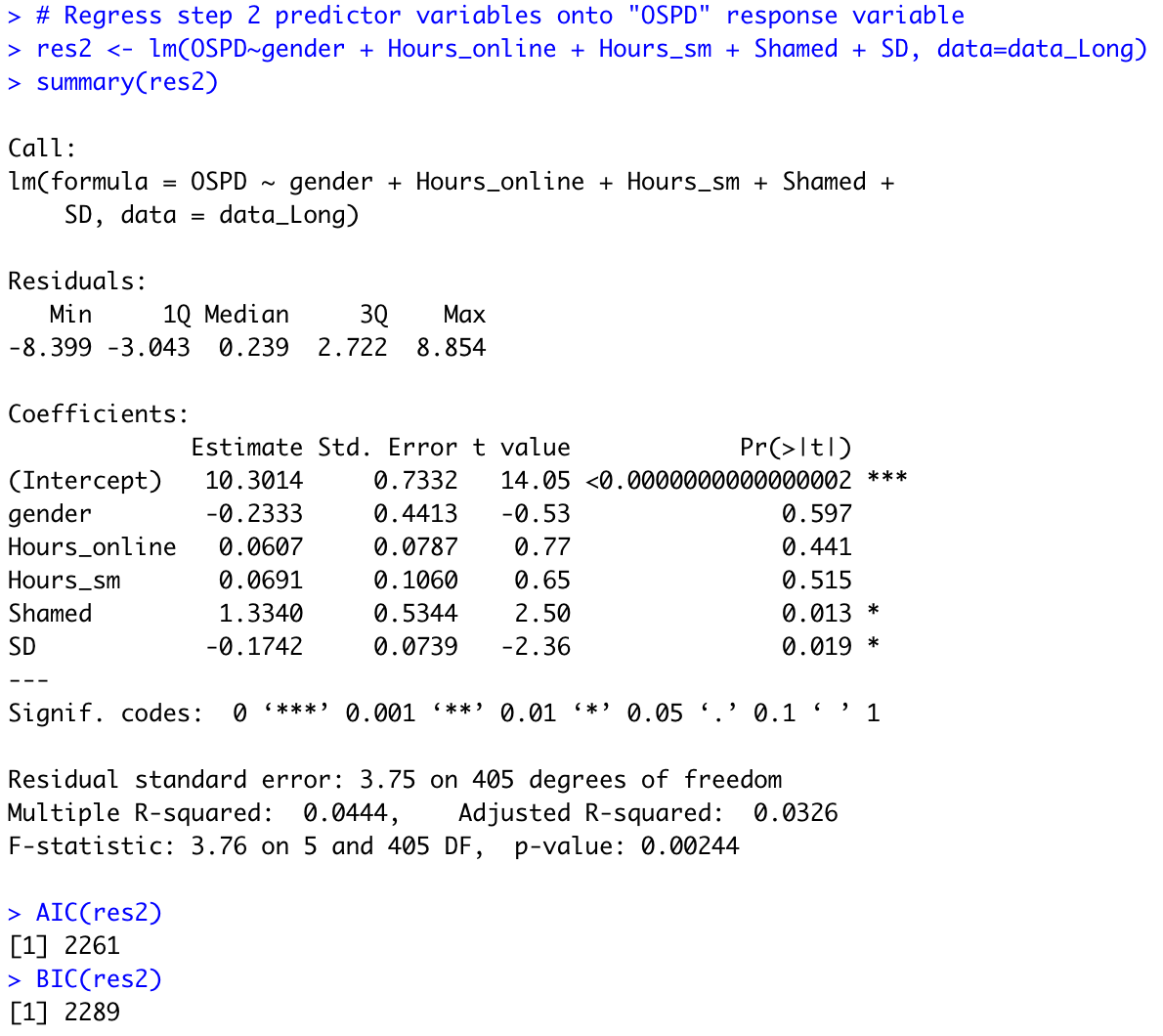
**

**Step 3.**

**
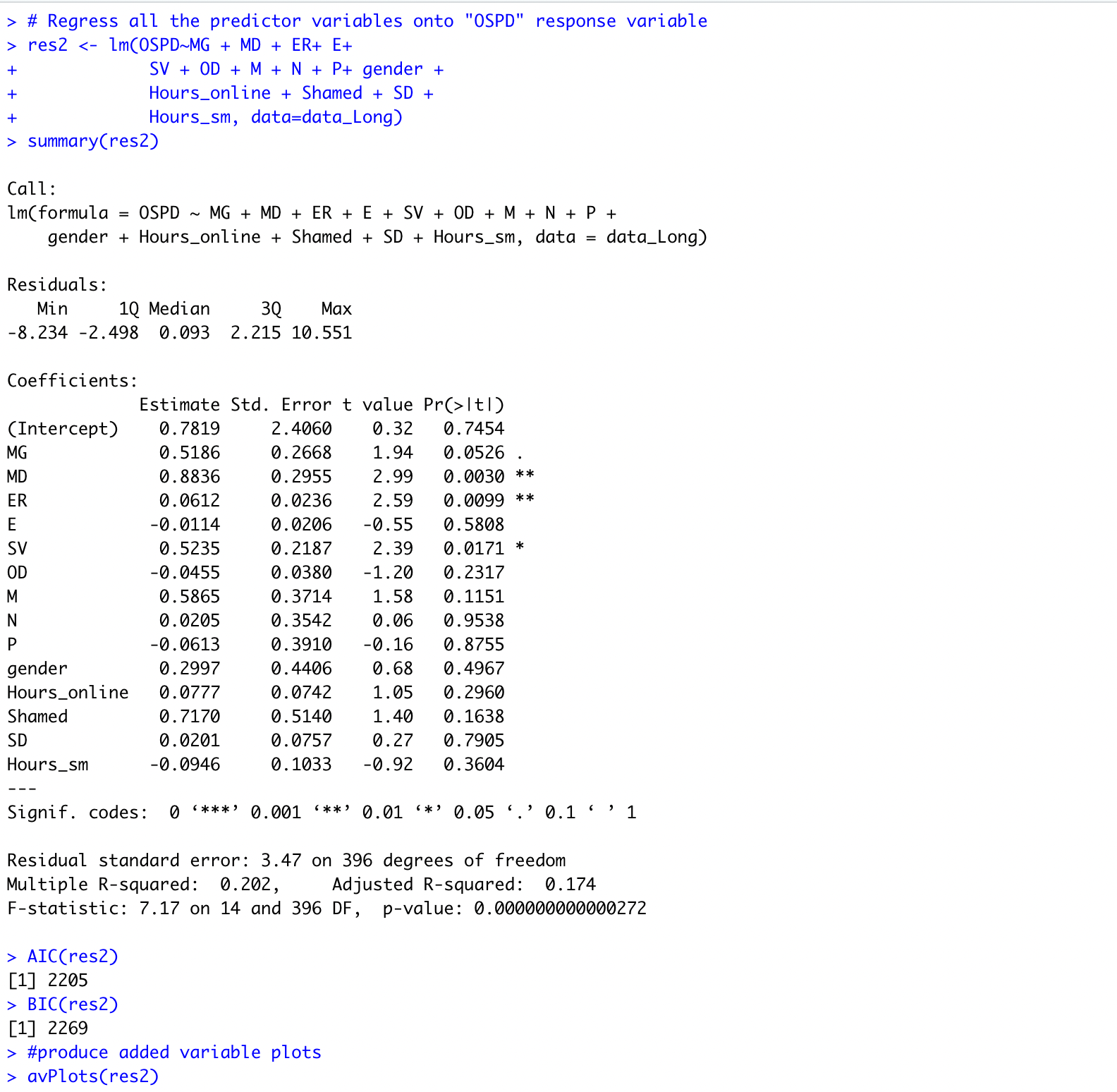
**

***Predicting online shaming perceived deservedness using Ridge regression in R***


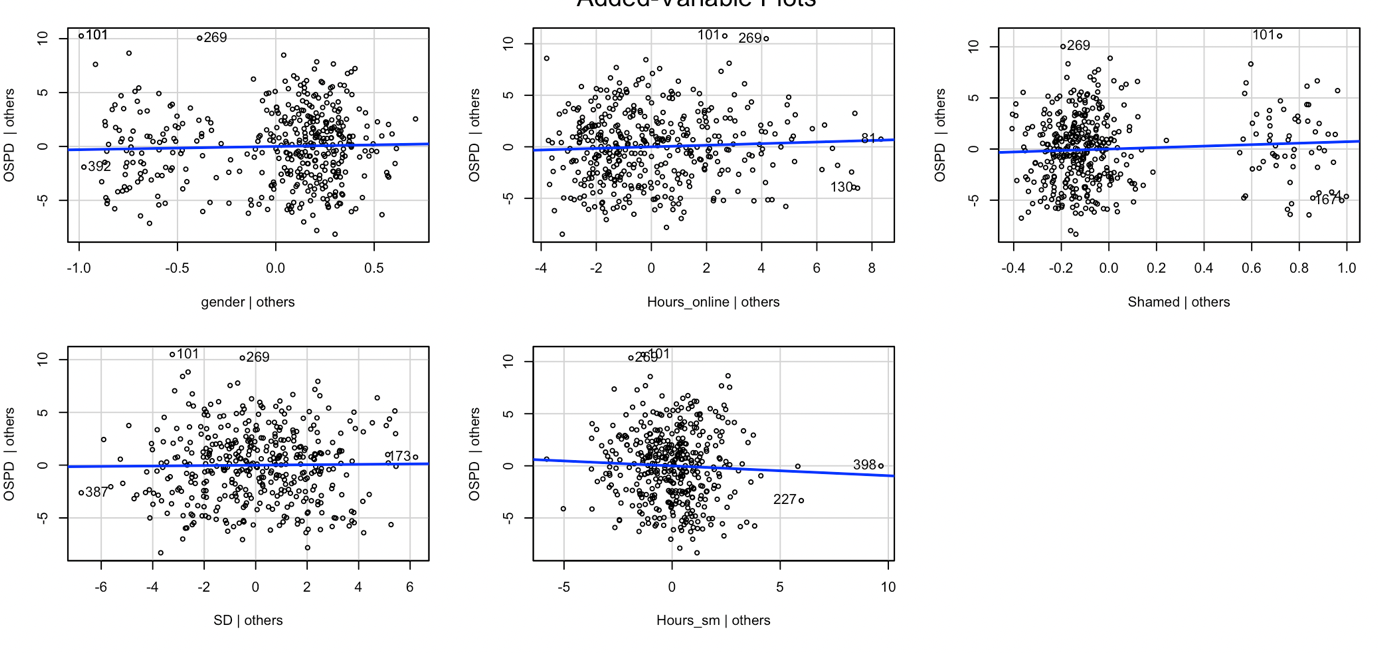


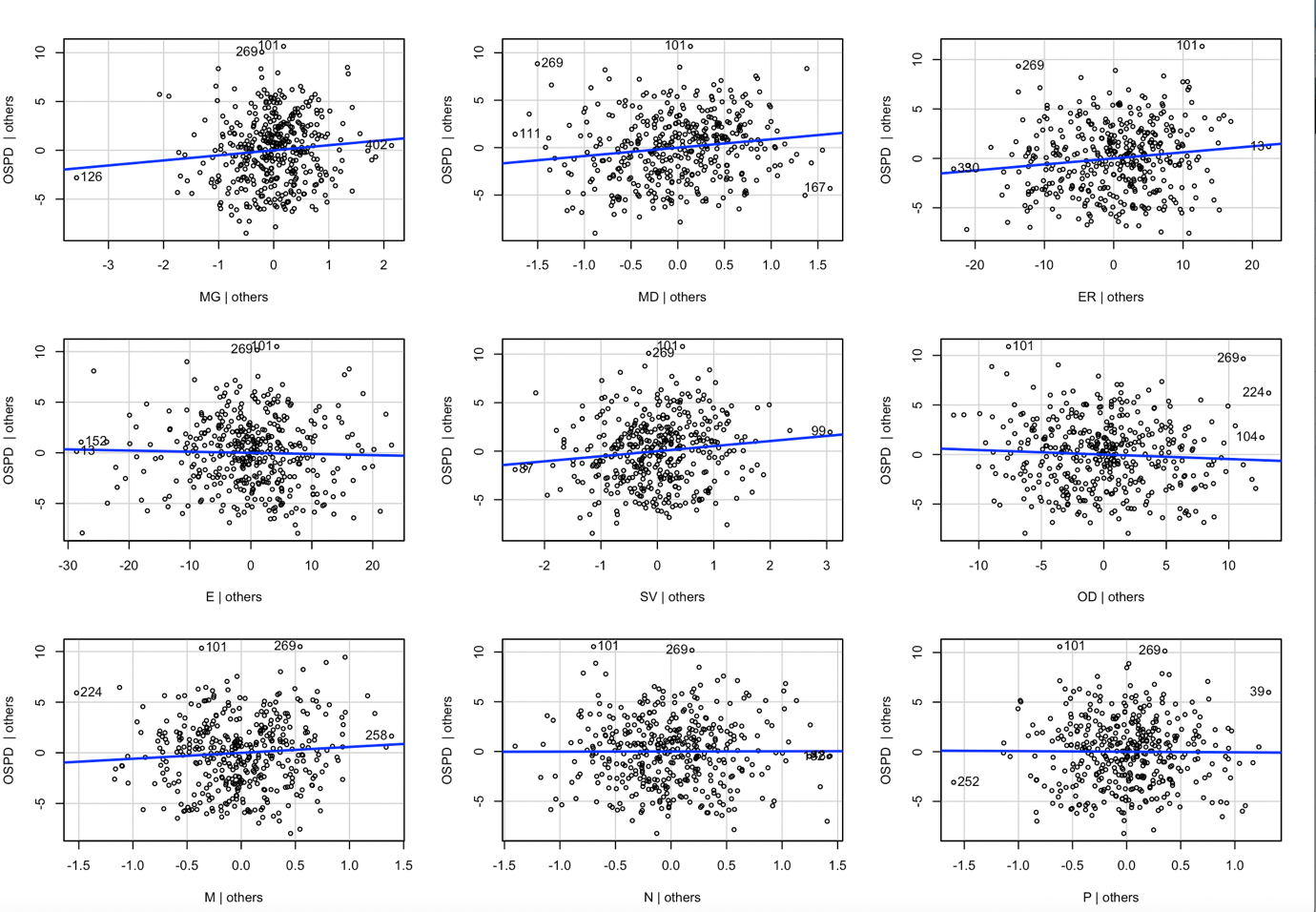


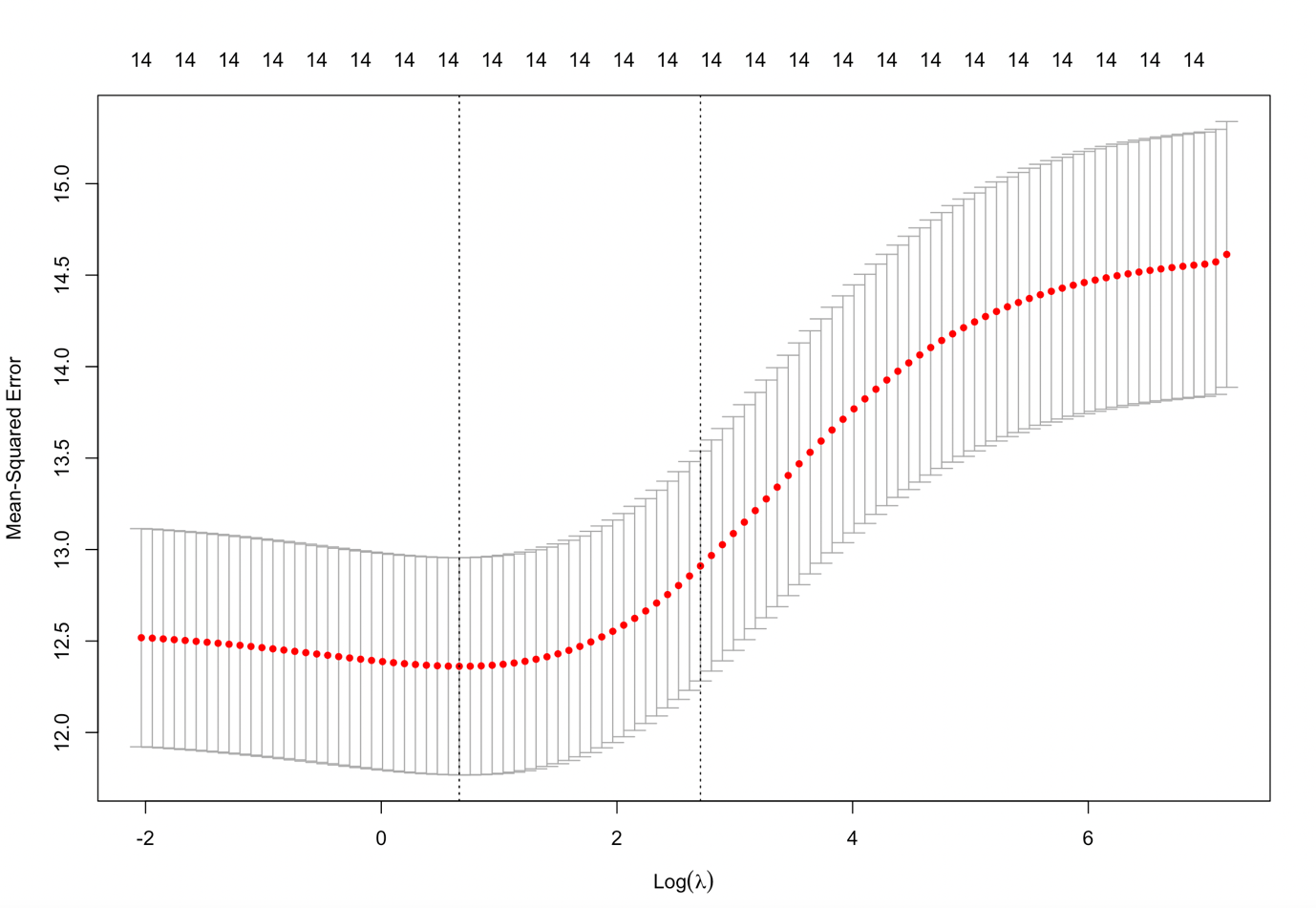


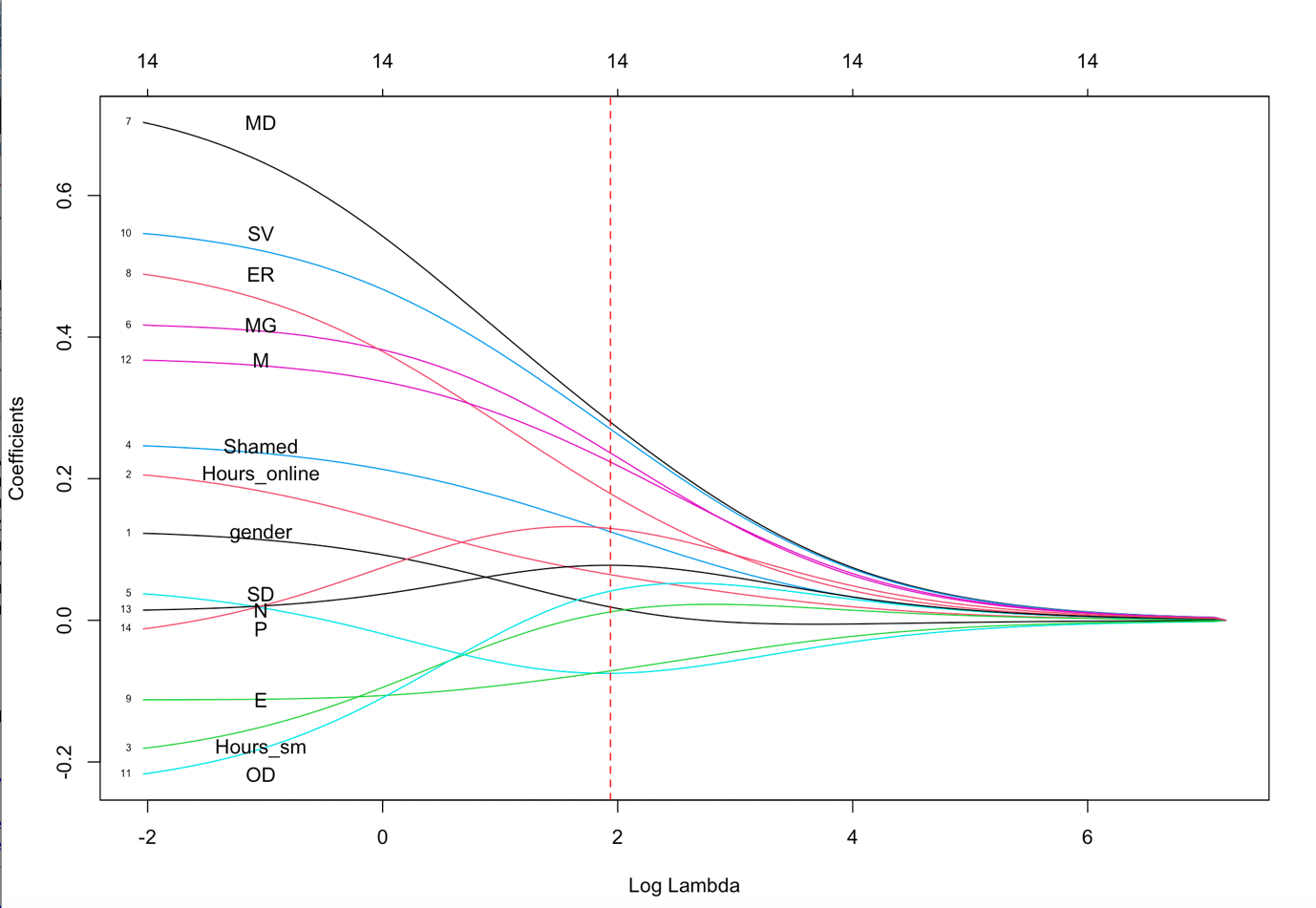


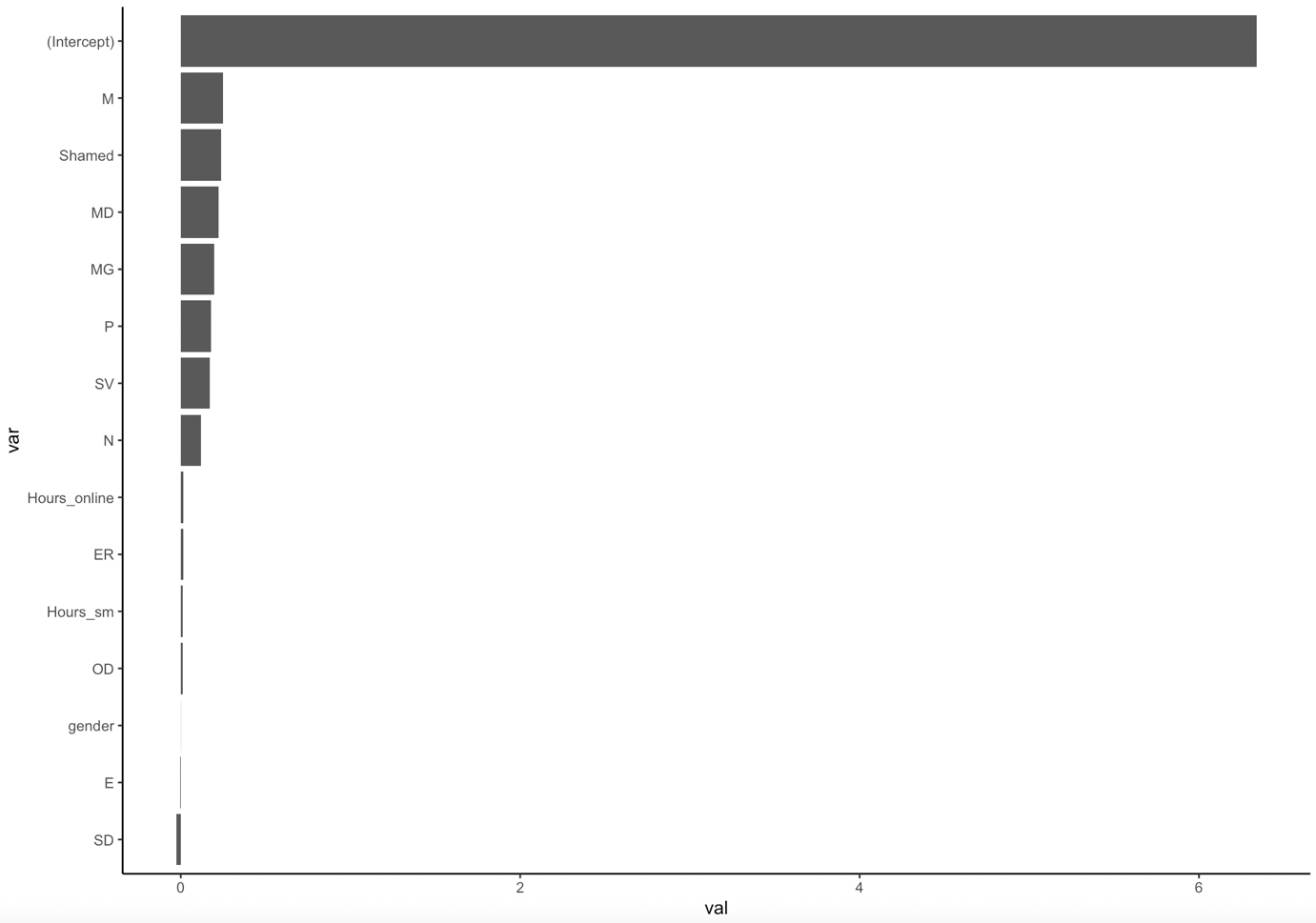


**
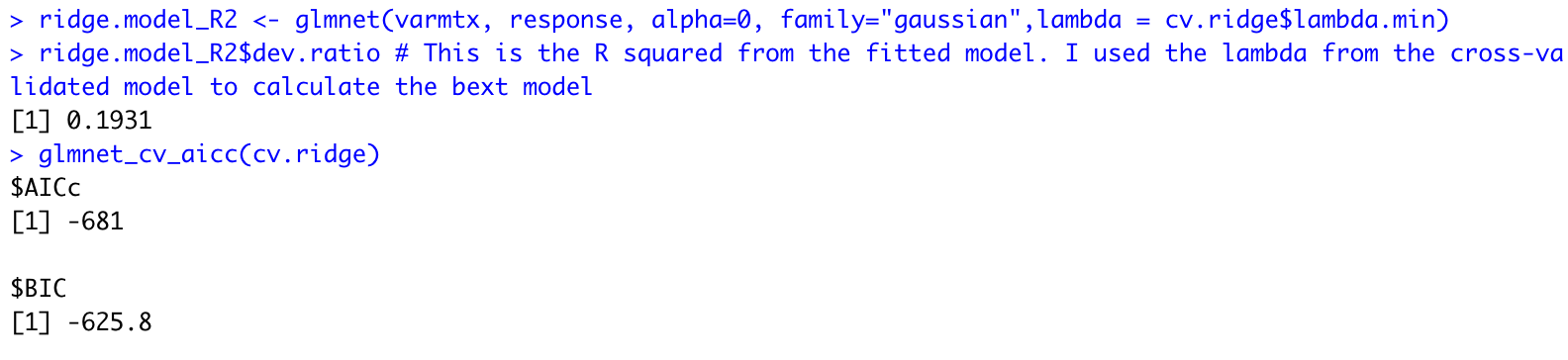
**

**
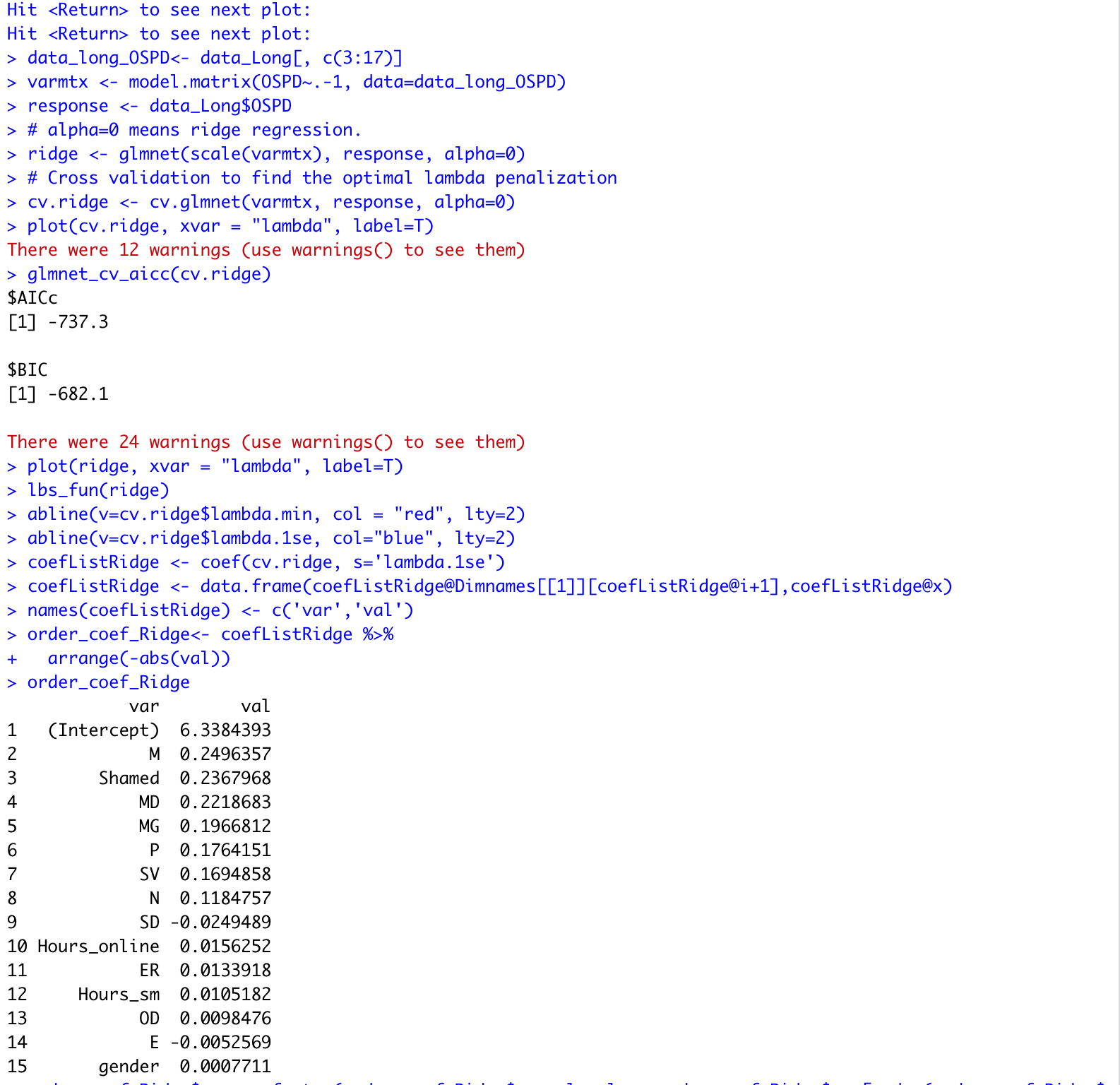
**
